# Supplementary material for: Single-Cell RNA Sequencing Reveals T Helper Cells Synthesizing Steroids De Novo to Contribute to Immune Homeostasis
Source: Cell Rep. 2014 May 9;7(4):1130–42. doi: 10.1016/j.celrep.2014.04.011 (PMC4039991; doi:10.1016/j.celrep.2014.04.011)
Supplement: Document S1. Supplemental Experimental Procedures, Figures S1–S5, and Table S1 [file mmc1.pdf]

Cell Reports, Volume *7*

Supplemental Information

Single-Cell RNA Sequencing Reveals T Helper Cells Synthesizing Steroids De Novo to Contribute to Immune Homeostasis

Bidesh Mahata, Xiuwei Zhang, Aleksandra A. Kolodziejczyk, Valentina Proserpio, Liora Haim-Vilmovsky, Angela E. Taylor, Daniel Hebenstreit, Felix A. Dingler, Victoria Moignard, Berthold Göttgens, Wiebke Arlt, Andrew N.J. McKenzie, and Sarah A. Teichmann

SUPPLEMENTAL INFORMATION

Figure S1

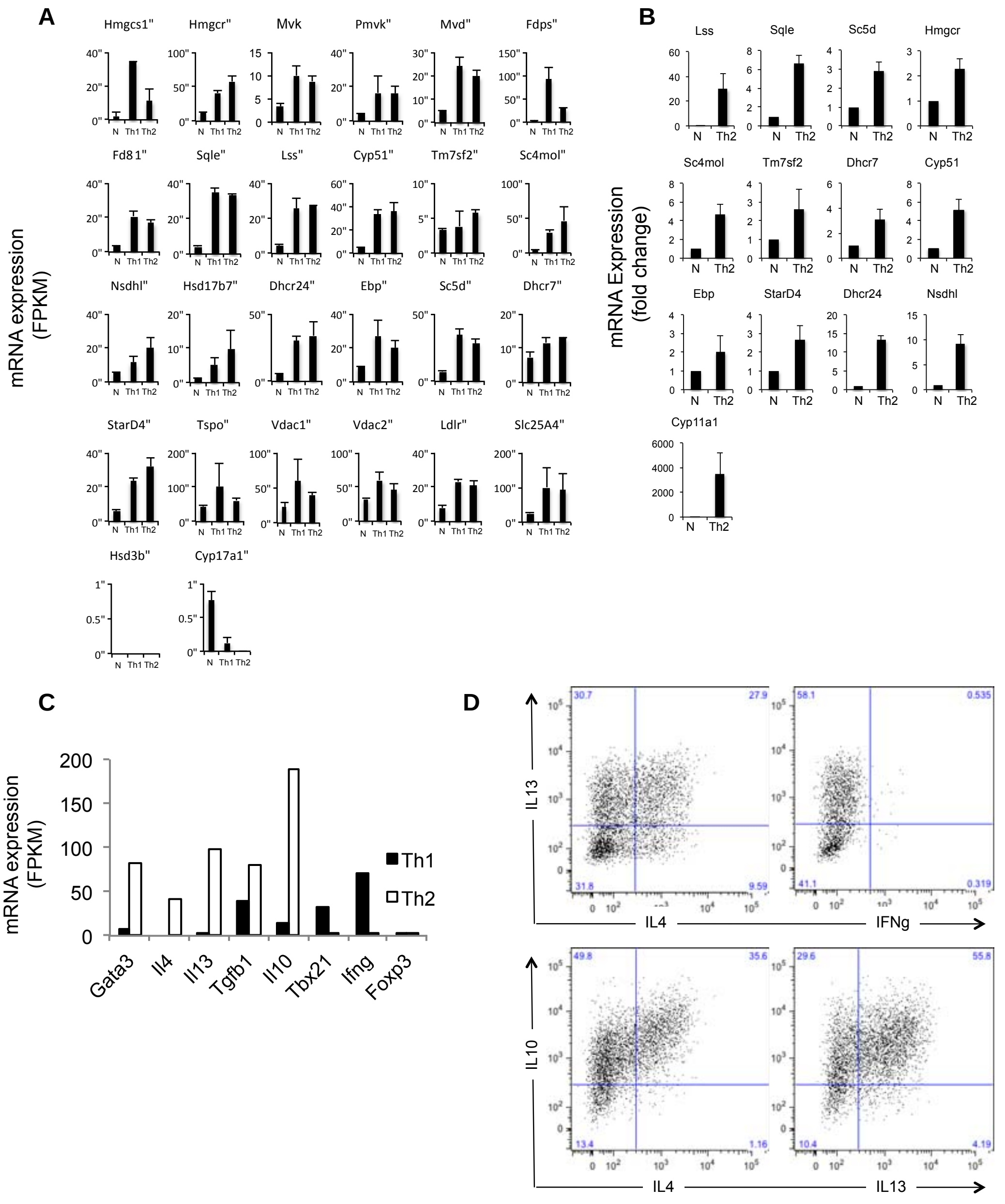

Figure S2

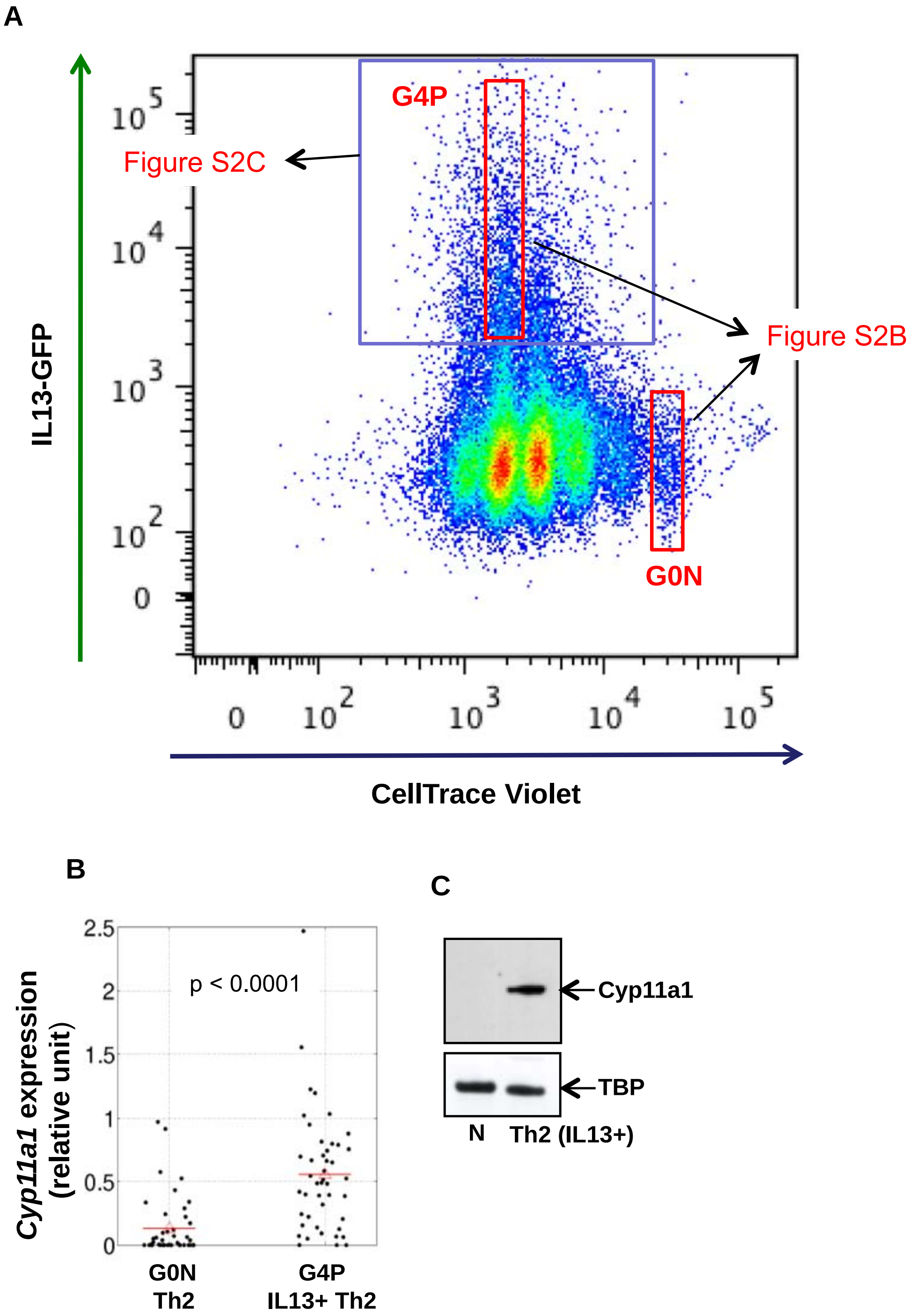

A
G4P
Figure S2C
Figure S2B
G0N
IL13-GFP
CellTrace Violet
0
10^2
10^3
10^4
10^5
B
p < 0.0001
Cyp11a1 expression (relative unit)
2.5
2
1.5
1
0.5
0
G0N Th2
G4P IL13+ Th2
C
Cyp11a1
TBP
N
Th2 (IL13+)

Figure S3

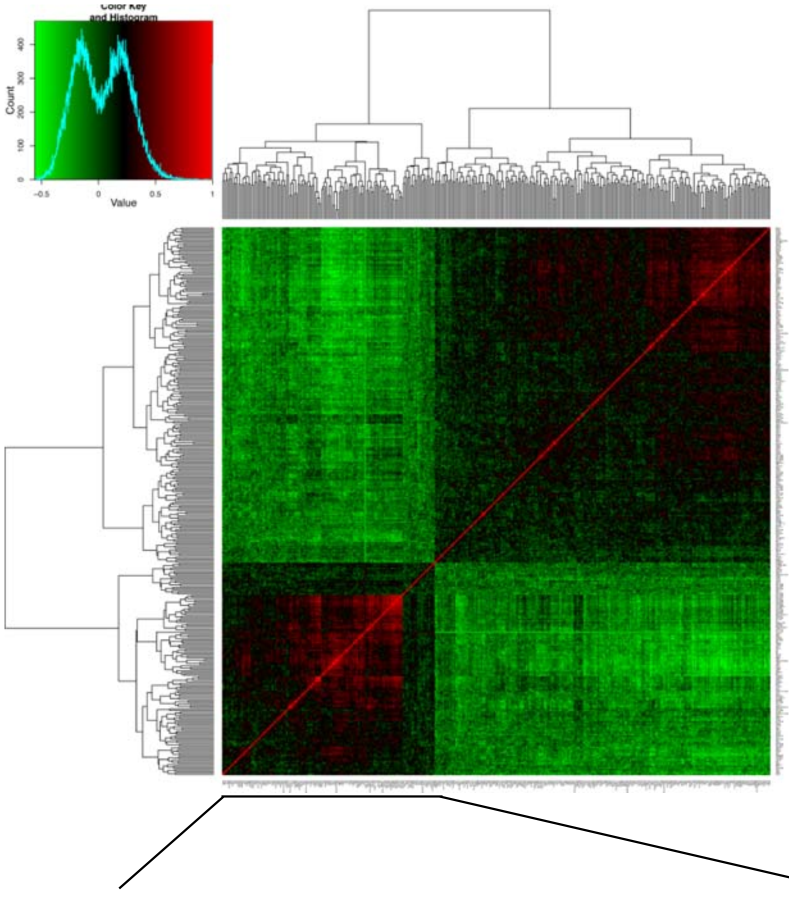

Gzma
Mcf2l
Tha1
Fanci
Gm16459
Rai2
Med13l
Syt2
Gipr
Lama5
Kremen2
Tsc22d4
Adamtsl4
Icos
Obfc1
Cysltr2
Itgb3
Fam46a
Gm13914
Gm13460
Dpm3
Sirt6
Gm11550
Gys1
Gm10767
Afg3l2
Acbd7
Myo10
Rfc3

Gpx8
Lst1
Pik3cg
Crygn
Gm13075
Anxa2
Ddit4
Atp6v0d1
Ostf1
0610010O12Rik
Anxa6
Gm13668
Mir5115
RP24−281K23.1
Tmsb10
Crip1
Gm14925
Gm9790
Eif4ebp1
AC124553.1
Ppp1r3b
Ndrg1
Arrb1
5033411D12Rik
Pold4
Tpt1
S100a1
Pkm
Gatsl3

Aldoart2
Aldoa
Aldoart1
Gm8659
Whsc1l1
Sell
Samhd1
Nfil3
Ctla2b
Ctla2a
Ecm1
Ly6c1
Ly6c2
9030619P08Rik
Ly6a
Basp1
Tgm2
Il12rb2
Mt1
Il24
Vim
Socs3
Il4ra
Cd24a
Tnfrsf9
Bsg
Fam162a
Gm9843

Ndufv3
Sdf4
Fxyd5
Cyp11a1
Gm11185
GAPDH
RP24−322E6.1
Gm7150
Gm3695
Pgam1−ps2
Tg
Gm7251
Gm11453
Serpinf1
RP23−429I18.1
Gm7293
Gm10293
Gm12416
Gm2308
AC168279.1
Gm12286
Gm5944
Gm13394
Gm13292
Gm9164
Gm15191
Gm10284
Gm16348

Trav7−3
Pkp2
Gm16225
Ppp1r18
Gm12586
Gm10658
Sdc3
Nucb2
Lrrtm3
Gcnt1
Hook2
9430020K01Rik
Tanc1
Zfyve16
Gm8877
Upf3a
Flywch1
D19Wsu162e
Arfgap3
Slc6a20b

Figure S4

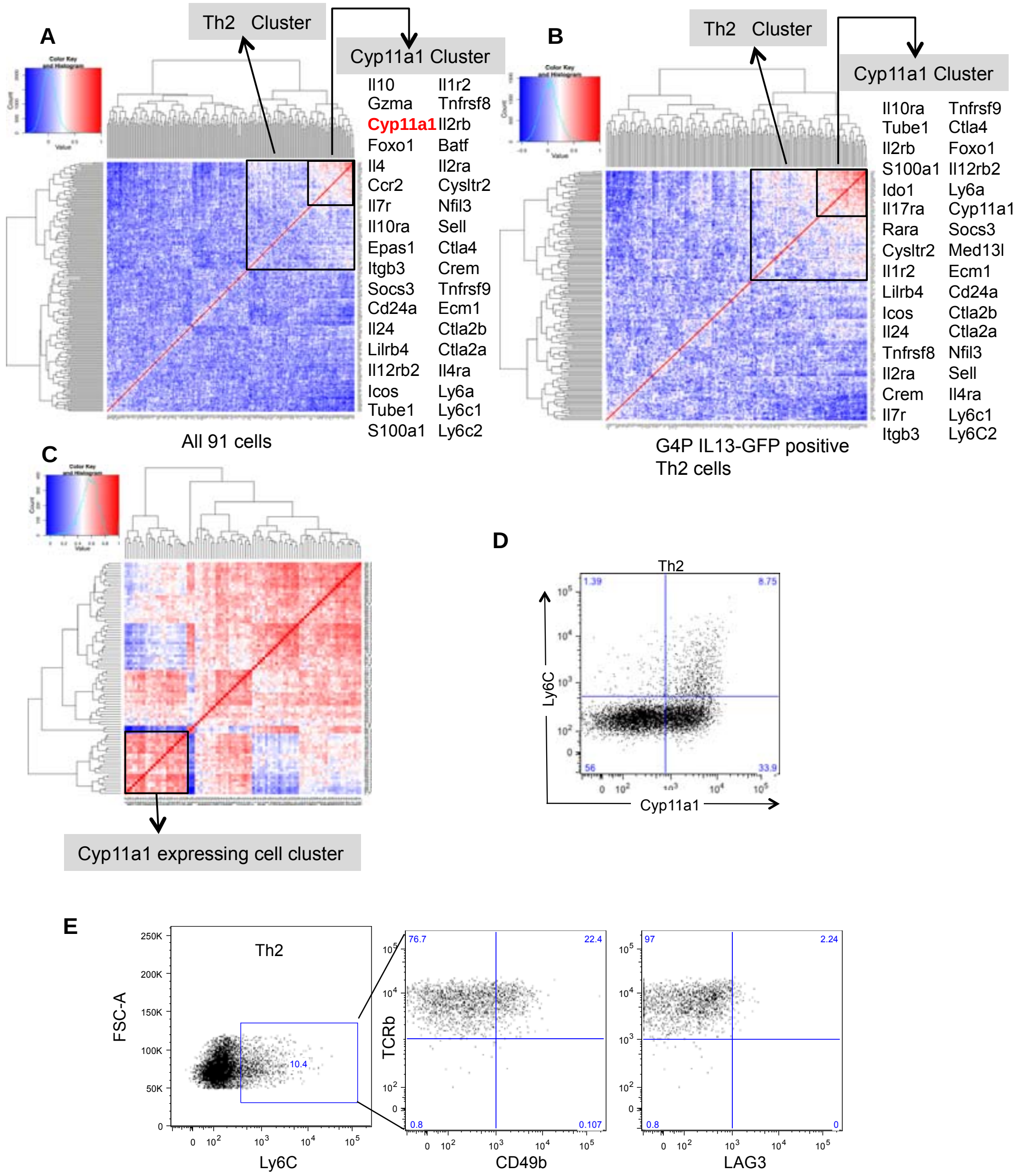
A
Th2 Cluster
Cyp11a1 Cluster
Il10 Il1r2
Gzma Tnfrsf8
Cyp11a1 Il2rb
Foxo1 Batf
Il4 Il2ra
Ccr2 Cysltr2
Il7r Nfil3
Il10ra Sell
Epas1 Ctla4
Itgb3 Crem
Socs3 Tnfrsf9
Cd24a Ecm1
Il24 Ctla2b
Lilrb4 Ctla2a
Il12rb2 Il4ra
Icos Ly6a
Tube1 Ly6c1
S100a1 Ly6c2
All 91 cells
B
Th2 Cluster
Cyp11a1 Cluster
Il10ra Tnfrsf9
Tube1 Ctla4
Il2rb Foxo1
S100a1 Il12rb2
Ido1 Ly6a
Il17ra Cyp11a1
Rara Socs3
Cysltr2 Med13l
Il1r2 Ecm1
Lilrb4 Cd24a
Icos Ctla2b
Il24 Ctla2a
Tnfrsf8 Nfil3
Il2ra Sell
Crem Il4ra
Il7r Ly6c1
Itgb3 Ly6C2
G4P IL13-GFP positive
Th2 cells
C
Cyp11a1 expressing cell cluster
D
Th2
Ly6C
Cyp11a1
1.39
8.75
56
33.9
E
Th2
FSC-A
Ly6C
10.4
TCRb
CD49b
76.7
22.4
0.8
0.107
LAG3
97
2.24
0.8
0

Figure S5

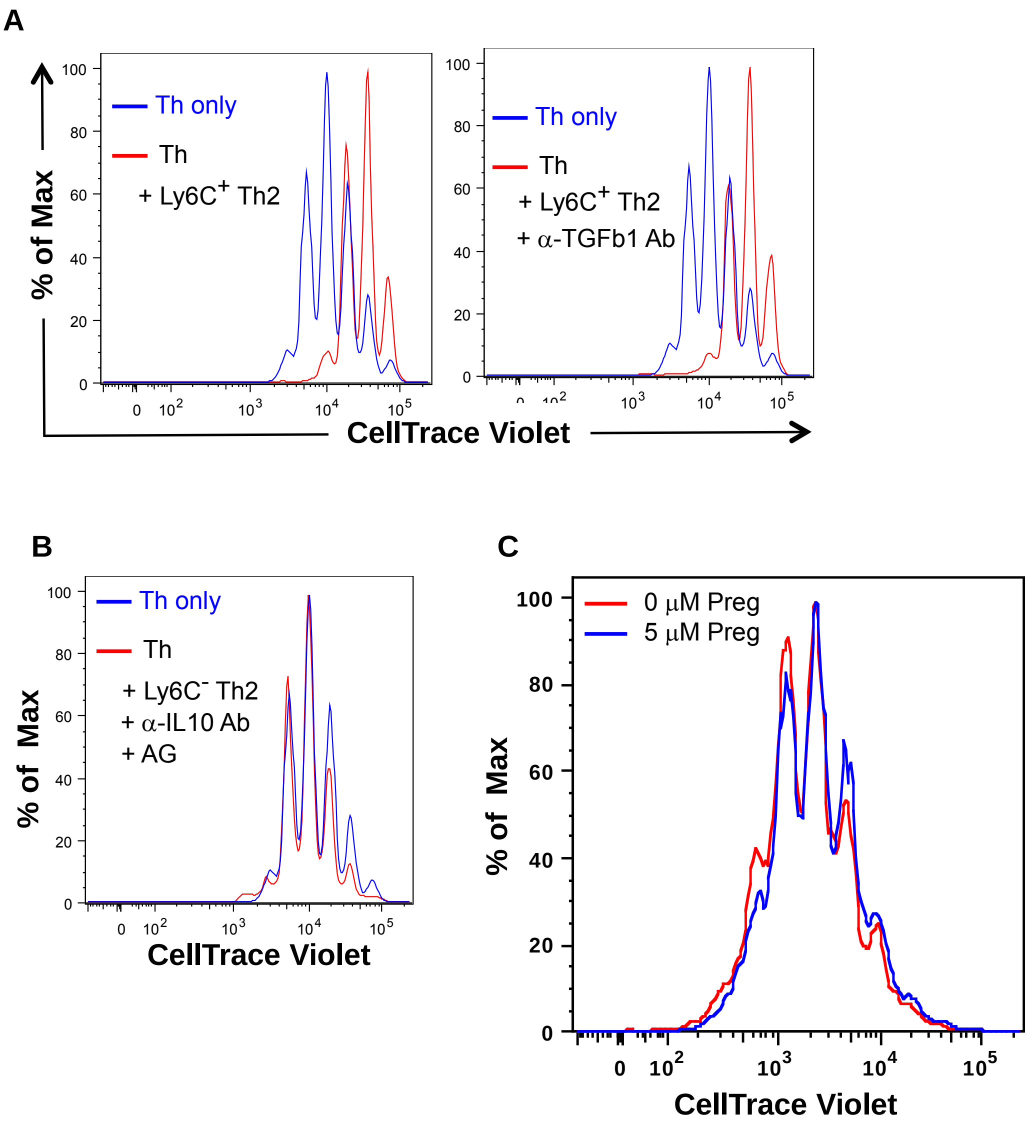
A
% of Max
CellTrace Violet
Th only
Th
+ Ly6C+ Th2
+ α-TGFb1 Ab
0
20
40
60
80
100
0
10^2
10^3
10^4
10^5
B
% of Max
Th only
Th
+ Ly6C- Th2
+ α-IL10 Ab
+ AG
CellTrace Violet
C
0 μM Preg
5 μM Preg
% of Max
CellTrace Violet

SUPPLEMENTAL FIGURE LEGENDS

Figure S1. Upregulation of a steroid synthesis pathway in Th2 cells *in vitro*

(A) Comparison of mRNA expression between Naïve (N), Th1 and Th2 cells

RNA-seq data for all genes involved in the steroid synthesis pathway was analyzed. Expression levels are shown as FPKM values. The histograms represent mean expression values for each mRNA obtained from two independent experiments. The values for naïve and Th2 are used in Figure 1 to generate color-coded maps. Please note that y-axis scales are variable, and expression levels at or below 1 FPKM are most likely simply stochastic gene expression as discussed in Hebenstreit et al., (2011).

(B) Quantitative PCR validation of selected genes in the steroid synthesis pathway. A set of genes observed to be upregulated in RNA-seq data were checked by qPCR. The bars represent mean expression values for each mRNA obtained from three independent experiments.

(C) RNA-seq gene expression profiles support the Th2-specific identity of cell culture. mRNA expression levels of Th2-specific genes Gata3, IL4, IL13; Th1-specific genes Tbx21 (or Tbet), IFNg; and Treg specific gene Foxp3. Expression levels are FPKM values in black for Th1 culture conditions and white for Th2. FPKM values are means obtained from two independent experiments.

(D) Cytokine expression profile of *in vitro* polarized Th2 cells by FACS after reactivation by PDBU and ionomycin.

Figure S2. Upregulation of Cyp11a1 in Th2 cells

(A) Gating strategy used to purify subpopulations of Th2 cells based on their cell division and IL13-GFP expression. Naïve T helper cells obtained from spleen of

IL13-GFP reporter mice were stained with CellTrace Violet dye and polarized for Th2 (3.5 days activation). The two subpopulations in the boxes were used in panels B and C below.

(B) Single-cell level comparison of Cyp11a1 mRNA expression between undifferentiated and differentiated Th2 cells. Cyp11a1 mRNA expression in single cells obtained from G0N and G4P subpopulations were detected by qPCR. Gene expression level was normalized to the mean of CD3e, Ubc, Hprt and Atp5a1 mRNA levels. Each dot represents a single cell. The two sets of values are significantly different (p-value 2.78×10^{-08}, Wilcoxon rank sum test).

(C) Splenic naïve Th cells from IL13-eGFP mice were activated-differentiated *in vitro* for Th2 polarisation. IL13-eGFP expressing mature Th2 cells were FACS sorted and analyzed for Cyp11a1 protein expression by Western blot.

Figure S3. Correlation heatmap of genes positively or negatively correlated with Cyp11a1 in single cell RNA-seq data. All genes with Spearman correlation coefficients with Cyp11a1 > 0.3 (133 genes) or < -0.3 (210 genes) were included for clustering based on the correlation coefficient. The bottom-left cluster corresponds to genes positively correlated with Cyp11a1 and the top-right cluster is negatively correlated genes.

Figure S4. Gene expression identity of Cyp11a1-expressing Th2 cells

(A,B,C) Cyp11a1 clusters with Th2 and suppressor genes and Cyp11a1+ cells cluster. (A) Hierarchical clustering on the Spearman correlation coefficient matrix of the 175 selected immunity genes (listed in Table S1) in all 91 cells. A large cluster

includes Th2 genes, and a more compact cluster includes Cyp11a1 and many suppressor genes.

(B) Hierarchical clustering on Spearman correlation coefficient matrix of the 175 genes in 52 G4P Th2 cells (4th generation and positive for IL13-GFP, Figure S4). A large cluster includes Th2 genes, and a more compact cluster includes Cyp11a1 with many suppressor genes.

(C) Hierarchical clustering on Spearman correlation matrix of cells based on 78 genes: 46 genes negatively correlated with Cyp11a1 (Spearman correlation < -0.35), and 32 genes positively correlated with Cyp11a1 (Spearman correlation > 0.35). The leftmost cluster (black box) of cells corresponds to those with high Cyp11a1 expression.

(D, E) Ly6C+ Th2 cells are steroidogenic and do not express type-1 regulatory (Tr1) markers LAG3 and CD49b

(D) *In vitro* polarized Th cells (3 days activated, 2 days resting) were FACS analyzed for Ly6C and Cyp11a1.

(E) *In vitro* polarized Th2 cells (3 days activated, 2 days resting) were analyzed for the presence of Ly6C, CD49b and LAG3 compared to the isotype control.

Figure S5.

(A) TGFb1 is not involved in Ly6C+ cell-mediated suppression. CellTrace Violet stained naïve Th cells were activated by anti-CD3e and anti-CD28 antibodies in the presence of FACS-sorted Ly6C+ or Ly6C- Th2 cells. The Ly6C+ cells were pre-treated with neutralizing anti-TGFb1 antibody (20μg/ml) for 24 hours. The histograms depicted are representative of 3 independent experiments with 3 mice in

each experiment. All culture conditions are as in Figure 5D. The first upper left panel of Figure 5D is reproduced here as the left panel for comparison.

(B) CellTrace Violet stained naïve Th cells ("responder cells") were activated by anti-CD3e and anti-CD28 antibodies in the presence of FACS sorted Ly6C- Th2 cells and anti-IL10 antibody and AG as indicated. The proliferation profile of the responder Th cells grown in the absence of Th2 (blue) was captured by FACS on the 3rd day of activation. This was compared to the proliferation profile of responder Th cells activated in the presence of Ly6C- Th2 (red). The Ly6C- cells were pre-treated with neutralizing anti-IL10 antibody (20μg/ml) or with aminoglutethimide (AG, 250μM) or with both, for 24 hours, and were used in the same culture conditions. The responder Th cell to Ly6C- Th2 cell ratio was 1:1. The panel is shown as a control for the same experimental condition when performed with Ly6C+ Th2 cells (as shown in Figure 5D, bottom row, right)

(C) No effect of pregnenolone on B cell proliferation. Proliferation of untreated and 5μM pregnenolone treated B cells was monitored by dye dilution in the same experiments described in Figure 6F. There is no significant difference between treated and untreated cells.

Table S1 List of 175 selected genes involved in immune response

Ahr	Cd86	Egr2	Il12a	Il5	Maf	Socs3
Asb2	Cd8b1	Egr3	Il12rb1	Il6	Med13l	Socs5
Batf	Cdkn1b	Epas1	Il12rb2	Il7	Nfatc1	Tanc2
Bcl6	Cebpb	F2r	Il13	Il7r	Nfatc2	Tbx21
Cblb	Cngb1	Foxo1	Il13ra1	Il9	Nfatc2ip	Tcf7
Ccl5	Crebbp	Foxp1	Il15	Inhba	Nfatc3	Tfcp2
Ccnjl	Crem	Foxp3	Il17ra	Irf1	Nfil3	Tgfb1
Ccr1	Csf1r	Gata3	Il17rb	Irf4	Nr1i2	Tgfb3
Ccr2	Csf2	Gfi1	Il18	Irf8	Nt5e	Tgfbi
Ccr3	Ctla2a	Gpr44	Il18bp	Itch	Ostf1	Tgfbr3
Ccr4	Ctla2b	Gzma	Il18r1	Itga2	Pcgf2	Tmed1
Ccr5	Ctla4	Gzmb	Il1r1	Itgae	Prdm1	Tmtc2
Ccr7	Cxcr1	Havcr2	Il1r2	Itgam	Prf1	Tnf
Ccr8	Cxcr3	Icam1	Il2	Itgb3	Ptgir	Tnfrsf14
Cd24a	Cxcr4	Icos	Il21	Jdp2	Ptprc	Tnfrsf18
Cd27	Cxcr5	Ido1	Il22	Junb	Rara	Tnfrsf25
Cd28	Cxcr6	Ido2	Il23a	Klrb1	Rnf128	Tnfrsf4
Cd38	Cyp11a1	Ifng	Il23r	Klrd1	Rora	Tnfrsf8
Cd3e	Cysltr2	Ifngr1	Il24	Lag3	Rorc	Tnfrsf9
Cd4	Ddit3	Ifngr2	Il27ra	Lif	Runx3	Tnfsf11
Cd40	Dgka	Igsf6	Il2ra	Lilrb4	Rxra	Tnfsf4
Cd40lg	Dpp4	Ikzf2	Il2rb	Lrrc32	S100a1	Tube1
Cd44	Dusp4	Il10	Il3	Ly6a	Sell	Tyk2
Cd68	Ebi3	Il10ra	Il4	Ly6c1	Socs1	Xbp1
Cd80	Ecm1	Il10rb	Il4ra	Ly6c2	Socs2	Yy1

Table S1. A list of genes characteristic of different cell types or involved in immune response (including effector and suppressor function). This list was compiled from the literature covering transcription factors, cell surface markers (receptors), cytokines and chemokines. 175 of these were found to be expressed at least at basal level in the 91 single cell transcriptomes. These genes were used to generate the clusters in Figures S6A and B.

Table S2. Genes with Spearman correlation coefficients with Cyp11a1 > 0.3 or s<-0.3, based on the single cell RNA-Seq data of all 91 Th cells (as shown in Figure 3A). For each gene, we annotate it by category: cytokine, transcription factor (TF), or surface marker.

Table S3. List of genes positively or negatively correlated with Cyp11a1 (Spearman correlation coefficient), based on the single cell RNA-Seq data of all 91 Th cells (G2N and G4P, as shown in Figure 3A). Genes with correlation coefficients > 0.2 or < -0.2 are shown, together with *p*-values.

Table S4. List of genes positively or negatively correlated with Cyp11a1 (Spearman correlation coefficient) based on the single cell RNA-Seq data of the 52 G4P Th2 cells (as shown in Figure 3A). Genes with Spearman correlation coefficients >0.2 or > -0.2 are shown, together with *p*-values.

EXTENDED EXPERIMENTAL PROCEDURES:

Abbreviations used in the Figure 1: ACoA: acetyl – CoA, AaCoA: Acetoacetyl-CoA, HCoA: HMG-CoA, M: mevalonate, M5P: mevalonate-5P, M5PP: mevalonate-5PP, IsPP: isopentyl-PP, FPP: farnesyl-PP, Squa: squalene, 23Ox: 2,3 oxydosqualene, Lan: lanesterol, 44Di: 4,4 dimethyl, cholesta-8,14,24-trienol, 14De: 14-demethyl-lanosterol, 4MZC: 4-methylzymosterol-carboxylate, 3K4M: 3-keto-4-methyl-zymosterol, 4MZ: 4-methylzymosterol, Zym: zymosterol, Cho8: cholesta-8,en-3beta-ol, Lath: lathosterol, DeC: 7-dehydro cholesterol, Cho7: cholesta-7,24-dien-3beta-ol, 7DeD: 7-dehydro-desmosterol, Des: desmosterol, OMM: outer mitochondrial membrane, IMM: inner mitochondrial membrane, Mito. Matrix: mitochondrial matrix.

T helper Cell Culture

Splenic naïve Th cells were purified by using the CD4+CD62L+ T Cell Isolation Kit II (Miltenyi Biotec) and were resuspended in IMDM, 10% FCS, 2 μM L-glutamine, penicillin, streptomycin and 50μM β-mercaptoethanol (purity > 95%). Alternatively CD4+CD25- naive T helper cells were purified by depletion of lymphocytes stained with FITC conjugated anti-CD8 (eBio Clone 53-6.7), anti-CD11b (eBio clone M1/70), anti-CD11c (eBio clone N418), anti-Ly6G (eBio Clone RB6-8C5), anti-CD19 (BD Bio Clone 1D3), and anti-CD25 (BD Bio Clone 7D4), and anti-FITC microbeads (Miltenyi Biotec) by MACS. Cells were seeded into anti-CD3e (1μg/ml, clone 145-2C11, eBioscience) and anti-CD28 (5μg/ml, clone 37.51, eBioscience) coated 96-well plates at a density of 250,000 to 500,000 cells/ml and a total volume of 200 μl. The medium contained the following cytokines and/or antibodies for the

different CD4+ subtypes. Th1: recombinant murine IL12 (10ng/ml, R&D Systems) and neutralizing anti-IL4 (5µg/ml, clone 11B11, eBioscience); Th2: recombinant murine IL4 (10ng/ml, R&D Systems) and neutralizing anti-IFN-γ (5µg/ml, clone XMG1.2, eBioscience); Th0: neutralizing anti-IL4 (5µg/ml, clone 11B11, eBioscience), neutralizing anti-IFN-γ (5µg/ml, clone XMG1.2, eBioscience). The cells were removed from the activation plate on day 4. Th1 and Th2 cells were cultured for another four days in absence of CD3 and CD28 stimulation to rest them. When required cells were restimulated with phorbol dibutyrate and ionomycin (both used at 500 ng/ml, both from Sigma) for 4 h in the presence of Monensin (2 µM, eBioscience) for the last 2 h after the resting phase (Hebenstreit et al., 2011).

RNA-seq, data generation, read mapping and expression level quantification

Poly-(A)+ RNA was purified from ~500 000 cells using the Oligotex kit (Qiagen). The manufacturer's protocol was slightly modified to include additional final elution steps resulting in a larger volume. After precipitation of RNA to concentrate it, first- and second-strand cDNA synthesis was performed using the Just cDNA kit (Stratagene), skipping the blunting step and directly proceeding to PCI extraction. Quality of the cDNA was tested by real-time PCR for a housekeeping gene. After this, the cDNA was sonicated for a total of 45 min using the Diagenode Bioruptor at maximum power settings, cycling 30 s sonications with 30 s breaks. Sequencing for 36 or 41bp was carried out on an Illumina GAII. The data were deposited at Gene Expression Omnibus (GEO, http://www.ncbi.nlm.nih.gov/geo/), accession numbers GSE28666 (Th2) and GSE31555 (all other sets). The reads for two biological replicates for each subtype were mapped using the spliced-read mapper TopHat version 1.2.0 (Trapnell et al., 2009) utilizing the Bowtie (version 0.12.7) short read

aligner (Langmead et al., 2009). The TopHat parameters used were "-r 328 –butterfly-search –solexal1.3-quals –p 8" and the "M. musculus, UCSC mm9" pre-built index file supplied by the TopHat website was used. To quantify expression levels, fragments per kilobase million (FPKM), reads per kilobase million (RPKM). Using Cufflinks version 0.9.3 (Trapnell et al., 2010), FPKM was calculated using the parameters "–reference-seq mm9.fa –GTF ucsc.gtf" where reference-seq enables bias correction, and –GTF looks for known transcripts in a gene transfer file (GTF) relative to the ucsc.gtf file. Cufflinks utilizes the maximum likelihood estimation method to calculate expression levels concurrently with various sequence and position specific biases which may affect expression levels, and produces a bias-adjusted measure (FPKM) of expression. A RefSeq based gene transfer file downloaded from the UCSC Genome Browser (accessed April 2011 from http://genome.ucsc.edu/ using the "table" function) was supplied to Cufflinks. Counts were determined from the TopHat SAM output using the HTSeq-counts program 0.4.7p4 (http://www-huber.embl.de/users/anders/HTSeq/doc/overview.html) using the parameters "–stranded=no --quiet." Counts were also used to calculate reads per kilobase million (RPKM) for single and paired end reads.

Quantitative PCR analysis

Total RNA was purified with the SV total RNA isolation kit (Promega) protocol. cDNA was prepared by annealing 500ng or 1μg RNA with oligo dT primers as per manufacturer's instructions (Transcriptor High Fidelity cDNA Synthesis kit, Roche). The cDNA samples were diluted 10 times with H_2O. 2 μl of diluted cDNA were used in 12μl qPCR reactions with appropriate primers and SYBR Green PCR Master Mix

(Applied Biosystems). Experiments were performed at least 3 times and data represent mean values +/- standard deviation.

Quantitative single-cell gene expression analysis

Single-cell gene expression analysis was performed using BioMark 96.96 Dynamic Array platform (Fluidigm, San Francisco, CA) and TaqMan Gene Expression Assays (Applied Biosystems, Carlsbad, CA). Single cells were sorted into 5µl of CellsDirect reaction mix and immediately stored in -80^0 C. Control wells containing 0 cells were included. On thawing, a mix containing 2.5µL gene specific 0.2x TaqMan gene expression assays (Applied Biosystems), 1.2 µL CellsDirect RT/Taq mix, and 0.3 µL TE buffer were added to each well. RT–PCR pre-amplification cycling conditions were: 50°C, 15min; 95°C, 2min; 22×(95°C, 15s; 60°C, 4min). Samples were diluted 1:5 in TE buffer and 6% were mixed with TaqMan Universal PCR Master Mix (Applied Biosystems). The sample mix and TaqMan assays were loaded separately into the wells of 96.96 Gene expression Dynamic Arrays (Fluidigm) in presence of appropriate loading reagents. The arrays were read in a Biomark analysis system (Fluidigm). ΔCt values were calculated in reference to the average of Atp5a1, Hprt1 and Ubc.

Single cell RNA sequencing and analysis:

Cell capture and library preparation for mouse cells using the Fluidigm C1 system

In vitro generated CellTrace Violet stained Th2 cells from 4th day of activation were FACS sorted to purify 4th generation IL13-GFP positive (G4P) and 2nd generation IL13-GFP negative (G2N), and mixed in equal proportion. 2000 cells were loaded onto a 10-17 micron C1 Single-Cell Auto Prep IFC, Fluidigm and cell capture was performed according to the manufacturer’s instructions. The capture efficiency was

inspected using a microscope, and there were single cells in 93 positions and two cells in 3 positions. These 3 positions were noted and subsequently the data from these cells were removed from analysis. Upon capture, reverse transcription and cDNA preamplification were performed in the 10-17 micron C1 Single-Cell Auto Prep IFC, Fluidigm using the SMARTer PCR cDNA Synthesis kit (Clontech) and the Advantage 2 PCR kit. 1 µl of the ERCC Spike-In Control Mix (Ambion) in a 1:400 dilution in C1 Loading Reagent was added to the Lysis Mix. cDNA was harvested and diluted to a range 0.1-0.3 ng/µl and Nextera libraries were prepared using the Nextera DNA Sample Preparation Kit and the Nextera Index Kit (Illumina) following the instructions in the Fluidigm manual "Using the C1TM Single-Cell Auto Prep System to Generate mRNA from Single Cells and Libraries for Sequencing". Libraries were pooled and paired end 75 bp sequencing was performed on 8 lanes of an Illumina HiSeq.

Mapping of reads and normalization for the mouse data set (91 cells)

Paired-end reads from 93 samples containing single cells were mapped simultaneously to the Mus musculus genome (Ensembl version 38.70) and the ERCC sequences using GSNAP (version 2013-02-05)17 with default parameters. Two cells were removed at this stage due to very low numbers of reads mapping to these libraries, leaving 91 cells in total. From here we proceeded as described previously in Online Methods Steps 8-11 (Brennecke et al., 2013).

SUPPLEMENTAL REFERENCES:

Brennecke, P., Anders, S., Kim, J.K., Kolodziejczyk, A.A., Zhang, X., Proserpio, V., Baying, B., Benes, V., Teichmann, S.A., Marioni, J.C., *et al.* (2013). Accounting for technical noise in single-cell RNA-seq experiments. Nature methods.

Hebenstreit, D., Fang, M., Gu, M., Charoensawan, V., van Oudenaarden, A., and Teichmann, S.A. (2011). RNA sequencing reveals two major classes of gene expression levels in metazoan cells. Molecular systems biology *7*, 497.

Langmead, B., Trapnell, C., Pop, M., and Salzberg, S.L. (2009). Ultrafast and memory-efficient alignment of short DNA sequences to the human genome. Genome biology *10*, R25.

Trapnell, C., Pachter, L., and Salzberg, S.L. (2009). TopHat: discovering splice junctions with RNA-Seq. Bioinformatics *25*, 1105-1111.

Trapnell, C., Williams, B.A., Pertea, G., Mortazavi, A., Kwan, G., van Baren, M.J., Salzberg, S.L., Wold, B.J., and Pachter, L. (2010). Transcript assembly and quantification by RNA-Seq reveals unannotated transcripts and isoform switching during cell differentiation. Nature biotechnology *28*, 511-515.
