# Supplementary material for: Single-Cell RNA Sequencing Reveals T Helper Cells Synthesizing Steroids De Novo to Contribute to Immune Homeostasis
Source: Cell Rep. 2014 May 9;7(4):1130–42. doi: 10.1016/j.celrep.2014.04.011 (PMC4039991; doi:10.1016/j.celrep.2014.04.011)
Supplement: Document S2. Article plus Supplemental Information [file mmc5.pdf]

OPEN ACCESS
CellPress

Cell Reports
Article

Single-Cell RNA Sequencing Reveals T Helper Cells Synthesizing Steroids De Novo to Contribute to Immune Homeostasis

Bidesh Mahata,[1,2,3,*] Xiuwei Zhang,[1] Aleksandra A. Kolodziejczyk,[3] Valentina Proserpio,[1,2,3] Liora Haim-Vilmovsky,[1,2,3] Angela E. Taylor,[4] Daniel Hebenstreit,[2,5] Felix A. Dingler,[2] Victoria Moignard,[6] Berthold Göttgens,[6] Wiebke Arlt,[4] Andrew N.J. McKenzie,[2] and Sarah A. Teichmann[1,3,*]
[1]EMBL-European Bioinformatics Institute, Wellcome Trust Genome Campus, Hinxton, Cambridge CB10 1SD, UK
[2]MRC Laboratory of Molecular Biology, Francis Crick Avenue, Cambridge Biomedical Campus, Cambridge CB2 OQH, UK
[3]Wellcome Trust Sanger Institute, Wellcome Trust Genome Campus, Hinxton, Cambridge CB10 1SA, UK
[4]Centre for Endocrinology, Diabetes, and Metabolism, School of Clinical and Experimental Medicine, University of Birmingham, Birmingham B15 2TT, UK
[5]School of Life Sciences, The University of Warwick, Coventry CV4 7AL, UK
[6]Department of Haematology, Cambridge Institute for Medical Research and Wellcome Trust and MRC Cambridge Stem Cell Institute, Hills Road, Cambridge CB2 0XY, UK
*Correspondence: bm11@sanger.ac.uk (B.M.), st9@sanger.ac.uk (S.A.T.)
http://dx.doi.org/10.1016/j.celrep.2014.04.011
This is an open access article under the CC BY license (http://creativecommons.org/licenses/by/3.0/).

SUMMARY

T helper 2 (Th2) cells regulate helminth infections, allergic disorders, tumor immunity, and pregnancy by secreting various cytokines. It is likely that there are undiscovered Th2 signaling molecules. Although steroids are known to be immunoregulators, de novo steroid production from immune cells has not been previously characterized. Here, we demonstrate production of the steroid pregnenolone by Th2 cells in vitro and in vivo in a helminth infection model. Single-cell RNA sequencing and quantitative PCR analysis suggest that pregnenolone synthesis in Th2 cells is related to immunosuppression. In support of this, we show that pregnenolone inhibits Th cell proliferation and B cell immunoglobulin class switching. We also show that steroidogenic Th2 cells inhibit Th cell proliferation in a Cyp11a1 enzyme-dependent manner. We propose pregnenolone as a "lymphosteroid," a steroid produced by lymphocytes. We speculate that this de novo steroid production may be an intrinsic phenomenon of Th2-mediated immune responses to actively restore immune homeostasis.

INTRODUCTION

An effective immune response is required for successful pathogen clearance. After clearance, the immune response must be terminated to restore immune homeostasis and avoid unwanted tissue damage or chronic inflammation (Viganò et al., 2012). T helper (Th) cells are central to the adaptive immune system. Depending upon the immunogen or allergen source (e.g., infection, commensal microorganism, or self-antigen), naive Th cells differentiate into several subtypes, including Th1, Th2, Th17, and iTreg, based on their cytokine profile and function (Zhu et al., 2010).

Upon extracellular pathogen infection (e.g., helminth infection), innate immune cells guide naive Th cells toward a Th2 phenotype. During type 2 immune responses, antigen experienced Th cells proliferate and differentiate toward the Th2 subtype and function through production of various effector cytokines, including interleukin-4 (IL-4), IL-5, IL-9, and IL-13, and at least two suppressor cytokines IL-10 and transforming growth factor (TGF)-β1 (Murphy et al., 2008). Th2 cells promote B cell class switching to IgE by expressing CD40 ligand (CD40L), IL-4, and IL-13 (Gould and Sutton, 2008). It is likely that there are undiscovered signaling molecules involved in type 2 immune responses. The active restoration or termination of a type 2 immune response is not well understood, though the importance of active termination has been discussed (Marrack et al., 2010; Viganò et al., 2012).

Specialized immune cells that act to suppress activation of the immune system and thereby maintain immune homeostasis and tolerance were documented many years ago (Gershon and Kondo, 1971) and extensively studied (Germain, 2008). The existence of suppressor Th2 cells has also been reported both in vivo and in vitro (Altin et al., 2012; Cua et al., 1995; Germain, 2008; Keino et al., 2001), but the mechanism of suppression is elusive and appears to be context dependent and manifold. Accepted suppression mechanisms by regulatory immune cells are expression of CTLA4 and secretion of IL-10 and TGF-β1 (Schmidt et al., 2012).

The immunoregulatory role of steroids has been extensively studied (Rhen and Cidlowski, 2005; Sakiani et al., 2013). It is exploited to treat patients where immunosuppression is required, such as organ transplantation, autoimmune diseases, allergic asthma, and inflammatory dermatitis (Barnes and Adcock, 2003; Gorter et al., 2010; Taylor et al., 2005). Steroid production is a multienzyme process by which cholesterol is

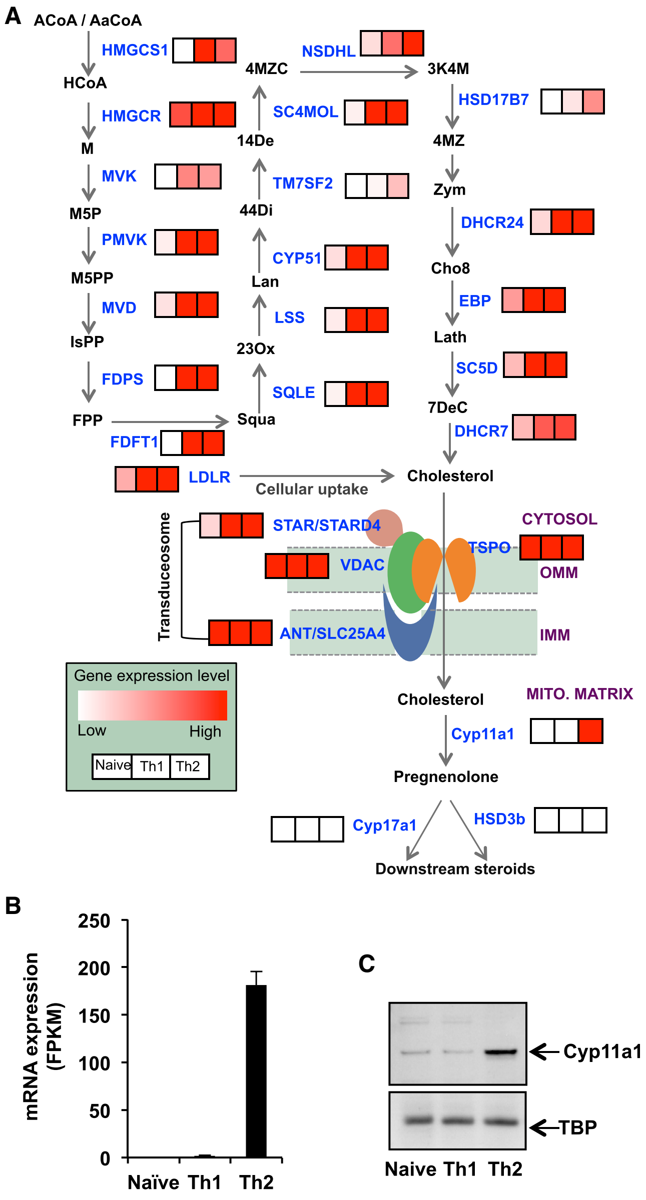

Figure 1. Upregulation of a Steroid Synthesis Pathway in Th2 Cells In Vitro

(A) Schematic representation of integrated steroid biosynthesis pathway with abbreviated metabolites (black) and protein factors (blue). Abbreviations are listed in the supplemental information. The first phase includes cholesterol biosynthesis and cholesterol uptake, the second phase is cholesterol transfer from the cytosol to the mitochondrial matrix, and the final phase includes steroid synthesis. mRNA expression of all the protein factors (blue) are shown as a color-coded map, where "white" indicates low expression and "red" indicates high expression level. FPKM (fragments per kilobase of transcript per million fragments mapped) values for each gene as obtained by RNA sequencing analysis were transformed to the color code (≤3 FPKM value as white and ≥15 FPKM value as maximum red). The comparison of the mRNA expression level between naive Th1 and Th2 cells is shown adjacent to the protein. The left-hand boxes represent color-coded expression levels in naive Th cells, middle is Th1, and right-hand boxes represent Th2 cells.

(B) Differential upregulation of Cyp11a1 mRNA. Cyp11a1 mRNA expression levels obtained by RNA-seq were compared in different Th cell types (naive [N], Th1, and Th2). Data presented are averages of two independent repeats. Error bars are SDs from the mean.

(C) Differential upregulation of Cyp11a1 protein. Comparison of Cyp11a1 protein expression in naive, Th1, and Th2 by western blotting.

converted to different steroid hormones (Miller and Auchus, 2011). After synthesis or receptor-mediated endocytosis, cholesterol is transported to the mitochondria through the transduceosome, a multisubunit protein complex composed of voltage-dependent anion channels (VDAC), translocator protein (TSPO), and Star-domain containing protein(s) (Midzak et al., 2011). Cholesterol synthesis and cellular uptake of cholesterol is necessary to support the de novo steroid biosynthesis.

After mitochondrial transfer, cholesterol is converted to pregnenolone, the first steroid hormone of the pathway, and precursor of all other steroids, by the enzyme Cyp11a1.

Our knowledge of steroid production is largely based on studies of the adrenal cortex, testicular Leydig cells, ovarian granulosa and theca cells, as well as placental syncytiotrophoblast cells (Miller and Auchus, 2011). Steroid production by other tissues ("local steroid production") has also been reported, particularly in the nervous system (Baulieu et al., 2001). Interestingly, immune-related tissues have also been found to have enzymatic activity for metabolizing steroids (Lechner et al., 2001; Vacchio et al., 1994). More interestingly, two prominent type 2 immune target tissues, gut and lung, were shown to convert the precursors to glucocorticoids upon type 2 immune activation (Cima et al., 2004; Hostettler et al., 2012). However, de novo steroid production from immune cells to regulate immune responses is unknown.

By comparing the transcriptomes of different Th subtypes, we discovered an upregulation of an entire steroid synthesis pathway in Th2 cells. We demonstrate de novo pregnenolone production by Th cells in vitro and in vivo and show pregnenolone-dependent inhibition of Th cell proliferation and immunoglobulin class switching in B cells. We speculate that the differentiation of steroidogenic subtype of Th2 cells is an intrinsic phenomenon of type 2 immune responses to restore immune homeostasis and will be relevant to the many molecular processes associated with allergies, asthma, pregnancy, and tumor immunity where Th2 cells are involved.

RESULTS

Transcriptomics Reveals Upregulation of a Steroid Synthesis Pathway in Th2 Cells

Analysis of our RNA sequencing data of murine naive and Th2 cells (NCBI Gene Expression Omnibus accession numbers: GSE28666 [Th2] and GSE31555 [naive, Th1]) revealed that Th2 lymphocytes express mRNAs encoding all the protein factors involved in the cholesterol synthesis, cholesterol uptake, mitochondrial transfer, and a steroid synthesis pathway (Figure 1). This pathway is shut down in naive Th cells but upregulated upon T cell receptor (TCR)- and IL-4-mediated activation and differentiation toward Th2 cells (Figures 1A and S1A). We validated the observed upregulation by quantitative PCR (qPCR) for selected genes (Figure S1B). The Th2 transcriptome

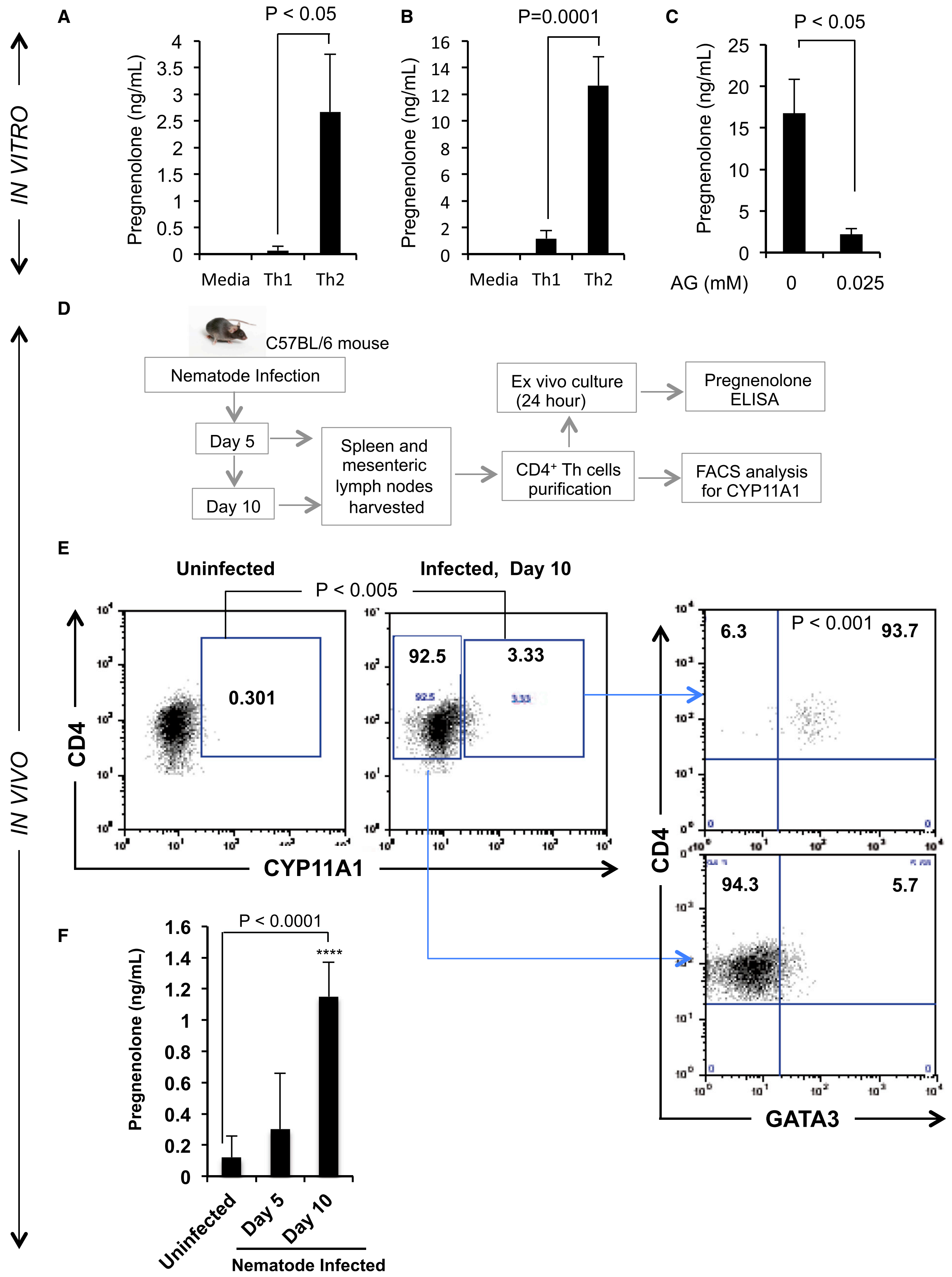

(legend on next page)

suggests the presence of pathway components up to the production of the steroid pregnenolone. The transcripts of enzymes downstream of pregnenolone, Cyp17a1 and Hsd3b, were undetectable (Figures 1 and S1A). The gene expression patterns of key transcription factors and cytokines validate the characteristics of Th1 and Th2 cell populations (Figures S1C and S1D). Note that all the genes involved in cholesterol biosynthesis and uptake were also upregulated in Th1 cells, but the steroid production pathway was incomplete in Th1 cells due to the absence of Cyp11a1 upregulation (Figures 1B and S1A).

Cyp11a1 Is Differentially Upregulated in Th2 Cells

Cyp11a1 is a key rate-limiting enzyme controlling the entry into the steroid synthesis pathways, by catalyzing the conversion of cholesterol to pregnenolone. The availability of the Cyp11a1 enzyme determines the initial step of de novo steroid production (Miller and Auchus, 2011). We analyzed and compared the Cyp11a1 mRNA expression level using both RNA sequencing (RNA-seq) (Figure 1B) and qPCR (Figure S1B, last panel) in different Th cell subtypes and found it to be differentially upregulated in Th2 cells at the population level.

To check mRNA expression at the single-cell level, naive Th cells from IL-13-eGFP mice were activated under conditions inducing Th2 differentiation. IL-13-eGFP-negative, undivided cells (G0N) and IL-13-eGFP-positive cells that had undergone four cycles of cell division (G4P IL-13^+ Th2) were fluorescence-activated cell sorted (FACS) as single cells (Figure S2A). mRNA expression levels in single cells showed an increased Cyp11a1 expression over the course of maturation, with a significantly higher mean Cyp11a1 expression in G4P IL-13^+ Th2 cells compared to G0N Th2 cells (Figure S2B).

Because mRNA expression does not always correlate with protein synthesis, we investigated Cyp11a1 abundance directly at the protein level. We noted differential upregulation of the protein in Th2 cells, compared to naive and Th1 (Figure 1C). When we determined Cyp11a1 protein levels in FACS-sorted homogeneous IL-13^+ Th2 cells, we found similar upregulation (Figure S2C).

Th2 Lymphocytes Produce Pregnenolone In Vitro

Cyp11a1-positive cells are considered as steroidogenic and expected to produce steroids de novo (Miller and Auchus, 2011). To examine steroid production, Th1 and Th2 culture supernatants were analyzed by liquid chromatography tandem mass spectrometry (LC-MS/MS) for quantitative detection of different steroids, including pregnenolone. We found that Th2 cells produce a significant amount of pregnenolone compared to a media-only control or Th1 cell supernatant (Figure 2A). We were unable to detect the presence of other steroids downstream of pregnenolone, such as progesterone or 17-hydroxypregnenolone (data not shown). Quantitative ELISA tests with the Th1 and Th2 cell supernatants validated this Th2-specific pregnenolone production (Figure 2B). Steroid production by Th2 cells was sensitive to aminoglutethimide (AG), an inhibitor of Cyp11a1 (Figure 2C), which suggests that production is actively catalyzed by Cyp11a1.

In vitro pregnenolone production suggests that Th cells may produce pregnenolone during a type 2 immune response in vivo, a hypothesis, which we set out to test using an in vivo helminth infection model.

Th2 Lymphocytes Produce Pregnenolone In Vivo

The nematode *Nippostrongylus brasiliensis* infection model in mice is used extensively to study the type 2 immune response in vivo (Camberis et al., 2003; Neill et al., 2010). During infection, the host mice mount both systemic and mucosal Th2 immune responses. IL-4-, IL-13-, and IL-5-producing GATA3^+ Th cells (Th2) cells have been shown to be indispensable for clearance of the parasites. Th2 cells induce B cells to produce IgE via secretion of IL-4, IL-13, and CD40L. This B cell switch toward IgE production is a critical event in type 2 immune response. The host defense eventually causes parasite expulsion from the intestine within 2 weeks (Fallon et al., 2002). We used this model to test Th2-mediated steroid production in vivo and analyzed pregnenolone expression during the height of worm burden at day 5 postinfection, and once clearance had occurred at day 10 postinfection.

Th cells were purified from spleen and mesenteric lymph nodes (MLNs) at postinfection day 5 and day 10 and examined for Cyp11a1 protein expression by FACS (Figure 2D). On day 10

Figure 2. The Steroid Synthesis Pathway in Th2 Cells Produces Pregnenolone: Steroid Production In Vitro during Th2 Polarization and In Vivo, in Response to Helminth Infection

(A) Quantitative detection of pregnenolone by LC-MS/MS. Naive Th cells were cultured in Th1 or Th2 activation-differentiation conditions. After 3 days of stimulation, cells were rested for 2 days with equal cell density. Cell supernatants were extracted for steroid profiling. Bars represent the mean pregnenolone concentration ±SEM. p values were calculated by unpaired two-tailed t test.

(B) Quantitative detection of pregnenolone by competitive ELISA. Th cells were stimulated for 3 days and rested for 3 days before supernatants were analyzed by competitive ELISA. Bars represent the mean concentration ±SEM from four independent experiments. p values were calculated using the unpaired two-tailed t test.

(C) Th2 supernatants were analyzed by ELISA for pregnenolone production as described in (B) with or without the presence of Cyp11a1 inhibitor, aminoglutethimide (AG). p values were calculated by unpaired two-tailed t test. Error bars are SDs from the mean.

(D) Schematic flowchart of in vivo experimental design. C57BL/6 mice were infected with nematode larvae (*Nippostrongylus brasiliensis)* for 10 days. On day 5 and day 10 postinfection, CD4^+ Th cells were purified from spleens and mesenteric lymph nodes (MLN). They were then immediately analyzed by FACS for Cyp11a1 expression, or ex vivo cultured for 24 hr for detection of pregnenolone production by ELISA.

(E) Cyp11a1 and GATA3 expression detected by FACS. Data shown were obtained from pooled samples of spleen and MLN of five mice and represent two experiments (n = 5 and n = 3 for each condition). p values were calculated using the unpaired two-tailed t test using data obtained from individual mice (n = [5 + 3] = 8).

(F) Pregnenolone concentration of ex vivo cultured Th cell supernatant was measured by quantitative ELISA. Results shown are mean pregnenolone concentration ±SD from both spleen and MLN. Experiments were performed twice with eight mice per condition. p values were calculated using unpaired two-tailed t test.

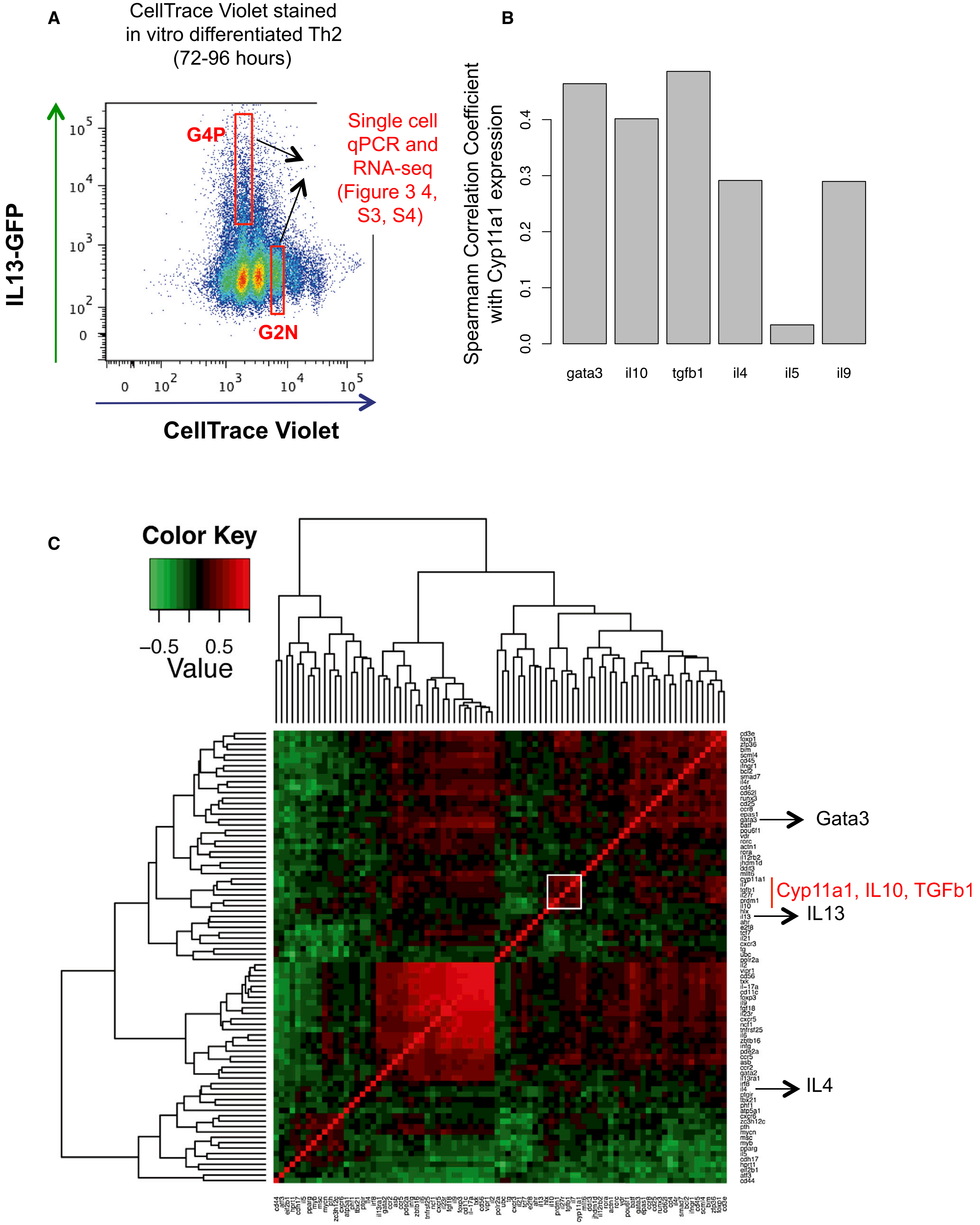

(legend on next page)

postinfection, Cyp11a1-expressing CD4$^+$ T cells were significantly enriched compared to the uninfected control (Figure 2E). A significant proportion (>90%) of CD4$^+$Cyp11a1$^+$ cells from postinfected day 10 mice coexpressed GATA3 compared to the CD4$^+$Cyp11a1$^-$ cells, which were <10% positive for GATA3 expression. These CD4$^+$GATA3$^+$Cyp11a1$^+$ cells represent a possible steroidogenic Th2 population during *N. brasiliensis* infection.

To test steroidogenic capacity, we cultured CD4$^+$ T cells ex vivo for 24 hr and analyzed cell supernatant for pregnenolone by ELISA. Th cells from postinfected day 10 mice secreted significantly higher amounts of pregnenolone compared to uninfected control mice (Figure 2F).

Upregulation of Cyp11a1 Is Associated with a Suppressor Phenotype

Th2 cells are not a homogeneous population; they produce different cytokine combinations in different contexts, and the functional outcome depends on the cytokine expression profile of the cell or cell population (Abbas et al., 1996; Prussin et al., 2010; Zhu and Paul, 2010). Thus, it is likely that there is further heterogeneity within this cell population, so we profiled the association of Cyp11a1 expression with cytokine expression at the single-cell level by qPCR (Figure 3A). This allowed us to explore the coassociation of different Th2 factors with Cyp11a1. The coexpression analysis of Cyp11a1 mRNA with different Th2 cytokines showed a higher correlation with suppressor cytokines (IL-10 and TGF-β1) than effector cytokines (IL-4, IL-5, and IL-9) (Figure 3B). The strong correlation between Cyp11a1 and the Th2-master regulator transcription factor GATA3 confirmed the Th2 identity of these cells (Figure 3B).

Upon hierarchical clustering on the matrix of pairwise Spearman correlation coefficients, Cyp11a1 clusters closely with TGF-β1, and IL-10, less closely with IL-13, IL-4, IL-5, and IL-9 (Figure 3C). This predicts a possible common or related regulatory mechanism governing Cyp11a1, and TGF-β1, and IL-10 expression, likely associated with suppression.

Single-Cell mRNA Sequencing Reveals the Gene Expression Identity of Steroidogenic Th2 Cells

To get further insight into the Cyp11a1 upregulation and a signature gene expression profile of Cyp11a1-producing cells, we sequenced mRNA of 91 single Th cells. Fifty-two cells were from the IL-13-GFP$^+$ fourth generation (G4P) and 39 were from the IL-13-GFP$^-$ second generation (G2N) (FACS gating shown in Figure 3A). From the single-cell transcriptomes, we identified Cyp11a1-correlated genes as those with a Spearman's rank correlation coefficient >0.3. This included several known genes involved in the type 2 immune response, and revealed numerous uncharacterized factors (Figure 4A). The genes ranked top of the list according to Spearman's correlation coefficient and p value are shown in Figure 4A in the following categories: cytokine, surface receptor, transcription factors, and others. Complete lists can be found in the Supplemental Information (Tables S2, S3, and S4).

Many Cyp11a1-correlated factors such as Nfil3, Crem, Gata3, IL-24, IL-4, IL-5, Gzma, Ecm1, and Itgb3 have been previously reported to be Th2 specific (Horiuchi et al., 2011). This indicates the Th2 origin of Cyp11a1-expressing cells. Expression of IL-4, GATA3, and Cyp11a1 mRNA at single-cell level is shown in Figure 4B.

Remarkably, the Cyp11a1-associated genes include many factors reported to be involved in immunosuppression, and development of regulatory or suppressor immune cells involved in restoration of immune homeostasis or immune tolerance. For example, Crem, Il10, TGFb1, Ctla2a, Ctla2b, Il2ra, Il2rb Il10ra, Ctla4, and Socs3 are well characterized for their role in immunosuppression. Nfil3 (Carey et al., 2013; Pletinckx et al., 2011), Med13l (Angus and Nevins, 2012), Foxo1 (Kerdiles et al., 2010; Ouyang et al., 2012), IL-24 (Myles et al., 2013), CD24 (Blair et al., 2010), and Tnfrsf9 (Eckstrum and Bany, 2011) have also been reported as immunosuppressive factors but are less characterized. Numerous other Cyp11a1-correlated factors, including the highest-correlated, Vimentin (Vim), are implicated in steroid biosynthesis and sterol trafficking (Kraemer et al., 2013) (Shen et al., 2012). Figure S3 shows the clustering heatmap of genes significantly positively or negatively correlated with Cyp11a1 based on their Spearman correlation matrix. The bottom-left cluster corresponds to genes positively correlated with Cyp11a1, and the top right is negatively correlated with Cyp11a1.

To gain further insight into the potential functional role of Cyp11a1-correlated genes in immune regulation, we selected 175 "immune" genes (Table S1). These genes are expressed at least at a basal level in the single-cell transcriptomic data set and are functionally characterized in immune responses. The hierarchical clustering heatmap with these selected genes revealed that Cyp11a1 clusters with many immunosuppressor genes. The Cyp11a1 cluster falls within a broader Th2-related gene cluster (Figures S4A and S4B).

To find the Cyp11a1-expressing subpopulation of cells, we take genes highly positively or negatively correlated with Cyp11a1 (Spearman correlation > 0.35 or ≤ 0.35). The heatmap based on the Spearman correlation coefficient matrix of these

Figure 3. Coexpression Pattern of Th2 Suppressor Cytokines with Cyp11a1 at Single-Cell Level

(A) Gating strategy used to purify subpopulations of Th2 cells based on their cell generation and IL-13-GFP expression. Naive Th cells obtained from spleen of IL-13-GFP reporter mice were stained with CellTrace Violet dye and polarized for Th2 (72–96 hr). The fourth-generation cells that expressed IL-13-GFP (G4P) and second-generation cells that did not express IL-13-GFP (G2N) were FACS sorted and used for single-cell gene expression analysis by qPCR and for single-cell RNA sequencing

(B) Spearman correlation coefficient (r) of Th2-associated genes with Cyp11a1 at single-cell level. Th2 cells from the G2N and G4P groups, as shown in (A) were FACS sorted as single cells. mRNA expression of different protein factors in a single cell were analyzed by single-cell qPCR.

(C) Hierarchical clustering of normalized mRNA expression data in single Th2 cell by qPCR for 73 selected genes. The clustering heatmap depicts patterns of coexpression among genes, where the green and red colors indicate the strength and direction of the gene-gene correlation (red meaning higher degrees of similarity, and green lower degrees of similarity). Hierarchical clustering was applied to group genes based on the similarity of their expression profile calculated by Spearman correlation across this data set. The gene cluster containing Cyp11a1 is boxed white, which contains Cyp11a1, IL-10, and TGF-β1.

OPEN ACCESS
CellPress

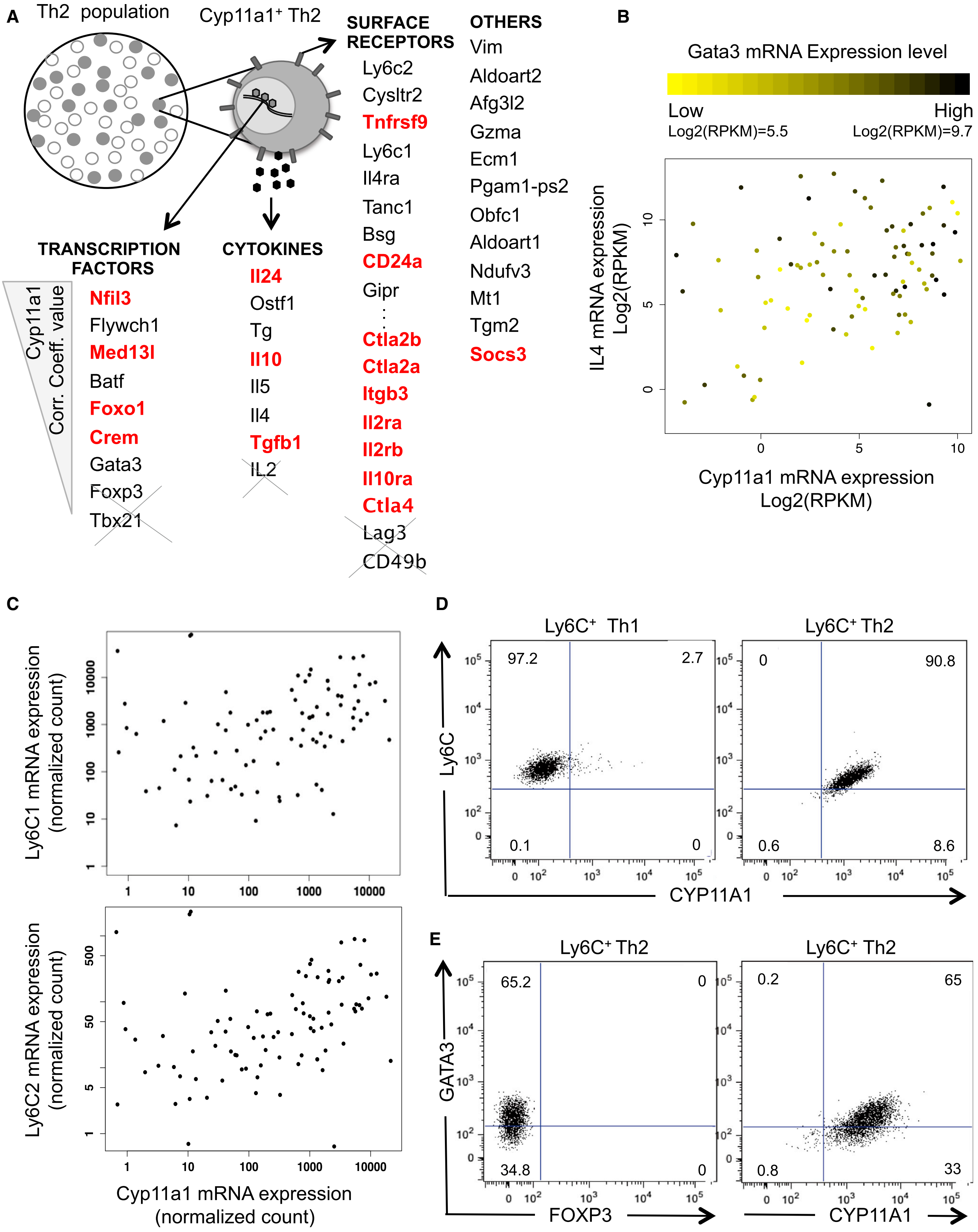

(legend on next page)

genes shows that most Cyp11a1-expressing cells cluster together as a single group (Figure S4C).

Ly6C1/Ly6C2-Expressing Th2 Cells Coexpress Cyp11a1 and Are Not Foxp3-Positive or Type 1 Regulatory T Cells

For functional analysis of Cyp11a1-expressing cells, it was critical to identify an appropriate cell surface marker to purify these cells. From the single-cell RNA sequencing analysis, we found a number of cell-surface receptors correlated to Cyp11a1 expression, of which Ly6C2 is at the top based on Spearman rank correlation coefficient (Figure 4A). Ly6C1 appears as fourth on this list (Figure 4A). mRNA expression of these two homologs at single-cell level is plotted against Cyp11a1 mRNA expression and shown in Figure 4C. We purified Ly6C$^+$ Th2 cells using an antibody that binds both homologs.

The FACS profile of in-vitro-generated Th2 cells show that nearly all the Ly6C$^+$ cells coexpressed Cyp11a1 (Figure S4D). After flow-cytometric purification, we found Ly6C$^+$ cells are extremely homogeneous for Cyp11a1 expression when purified from a Th2 population. In contrast, Ly6C$^+$ cells purified from a Th1 population do not express Cyp11a1 (Figure 4D). The Ly6C$^+$ Th cells also coexpress GATA3, but not FoxP3, indicating a Th2-type rather than Treg-type cell population (Figure 4D).

The single-cell RNA-seq analysis shows that these steroidogenic cells express IL-10 and TGF-β1 but not FoxP3, which raised a possibility that these cells are type 1 regulatory T cells (Tr1) (Gagliani et al., 2013). To check this, we carried out FACS analysis of LAG3, CD49b, and TCR-β in the Ly6C$^+$ Th2 population. As expected, nearly all cells were found to be TCR-β positive but < 25% CD49b$^+$ and < 5% LAG3$^+$ (Figure S4E); this means these cells are not Tr1 cells.

De Novo Steroid-Producing Th2 Cells Are Immunosuppressors

A number of steroids have been shown to suppress immune responses through inhibition of effector immune cell proliferation, differentiation, and/or by inducing cell death (Rhen and Cidlowski, 2005; Sakiani et al., 2013; Taylor et al., 2005). In Figures 3B and 4A, we show a correlation between Cyp11a1 expression and the suppressor cytokines IL-10 and TGF-β1. This led us to hypothesize that upon type 2 immune induction, activated Th2 cells produce pregnenolone as a signaling molecule to negatively regulate the immune effector response as a way to re-establish immune homeostasis.

Two major effector events in type 2 immune response are (1) rapid Th cell proliferation after TCR activation to achieve sufficient numbers of Th2 cells and (2) B cell differentiation toward an IgE-secreting phenotype through class switch recombination.

Pregnenolone Inhibits In Vitro Th1 and Th2 Cell Proliferation

To test the effect of pregnenolone in Th cell proliferation, CD4$^+$ naive Th cells were stained with CellTrace Violet and incubated in Th1 or Th2 activation-differentiation conditions in presence or absence of pregnenolone for 3 days. The presence of pregnenolone in the in vitro Th1 or Th2 culture conditions significantly retards cell proliferation when compared with vehicle-only-treated conditions (Figures 5A and 5B). Division indices, the average number of divisions that a cell (present in the starting population) has undergone, are presented in Figure 5C.

Ly6C$^+$ Th2 Cells Suppress Th Cell Proliferation

We observed that nearly all Ly6C$^+$ cells are Cyp11a1$^+$ (Figures S4D and S4E). We also observed that pregnenolone inhibits Th cell proliferation (Figures 5A and 5B). These results raised the possibility that Ly6C$^+$ cells could suppress Th cell activation/proliferation.

To check the functional physiological relevance of pregnenolone production by Ly6C$^+$Cyp11a1$^+$ Th2 cells, we used them in classical suppression assays (Collison and Vignali, 2011). CellTrace-Violet-stained naive Th cells were used as responder cells. The responder cells proliferate with a mean division index (DI) of 2.17 (Figure 5D, blue histogram in all panels) when plated on an anti-CD3e/CD28-coated plate. Proliferation is significantly suppressed when cocultured with Ly6C$^+$ Th2 cells in a 1:1 ratio (mean DI = 1.17) (Figure 5D, red histogram in left upper panel). In contrast, addition of Ly6C$^-$ Th2 cells had little or insignificant effect on responder Th cell proliferation (mean DI = 2.1) (red histogram in upper middle panel).

The suppressed proliferation rate was partly but significantly recovered when Ly6C$^+$ Th2 cells were pretreated with 250 μM aminoglutethimide (AG) or anti-IL-10 neutralizing antibody (20 μg/ml) for 24 hr and used in suppression assays in the presence of 250 μM AG or anti-IL-10 Ab (20 μg/ml) (Figure 5D, red histogram in upper-right and lower-left panels, respectively). The proliferation was significantly recovered when Ly6C$^+$ Th2 cells were pretreated with the mixture of 250 μM AG and anti-IL-10 neutralizing antibody (20 μg/ml) for 24 hr and used in suppression assays in the presence of both AG (250 μM) and anti-IL-10 Ab (20 μg/ml) (Figure 5D red histogram lower right panel). The additive effect of recovery, when IL-10 was neutralized in addition to blocking steroidogenesis, indicates the direct suppression by Cyp11a1 activity (i.e., steroid production). When a similar experiment was performed in parallel using Ly6C$^-$ Th2 cells, we observed no changes compared to

Figure 4. Gene Expression Identity of Cyp11a1-Expressing Th2 Cells and Ly6C as a Surface Marker for Steroidogenic Cell Purification

(A) Schematic summary of Cyp11a1-correlated genes as obtained from mRNA sequencing analysis of 91 single Th2 cells (52 cells from G4P and 39 cells from G2N, as shown in the Figure 3A). Genes are listed according to their correlation coefficient value, higher to lower, top to bottom in each category. Genes previously reported to be involved in immunosuppression are red and genes not coexpressed are crossed out. RNA sequencing and analysis are as described in Figures S3 and S4 and Tables S2, S3, and S4.

(B) IL-4 mRNA expression level was plotted against Cyp11a1 mRNA expression as obtained from single-cell RNA sequencing. Each dot represents an individual cell. The color of a dot varies from yellow to black, representing the mRNA expression level of Gata3 in the cell.

(C) Normalized mRNA expression level of Ly6C1 and Ly6C2 plotted against Cyp11a1 mRNA expression. Each dot represents an individual cell.

(D) Ly6C$^+$ cells were FACS sorted from in vitro differentiated Th1 (left panel) and Th2 (right panel) cell populations and FACS analyzed for Cyp11a1. Purified Th1 cells have a trace proportion of Cyp11a1$^+$ cells, whereas almost all purified Th2 cells are Cyp11a1$^+$.

(E) Ly6C$^+$ cells were FACS sorted from in vitro differentiated Th2 population and FACS analyzed for the expression of GATA3, FOXP3, and Cyp11a1

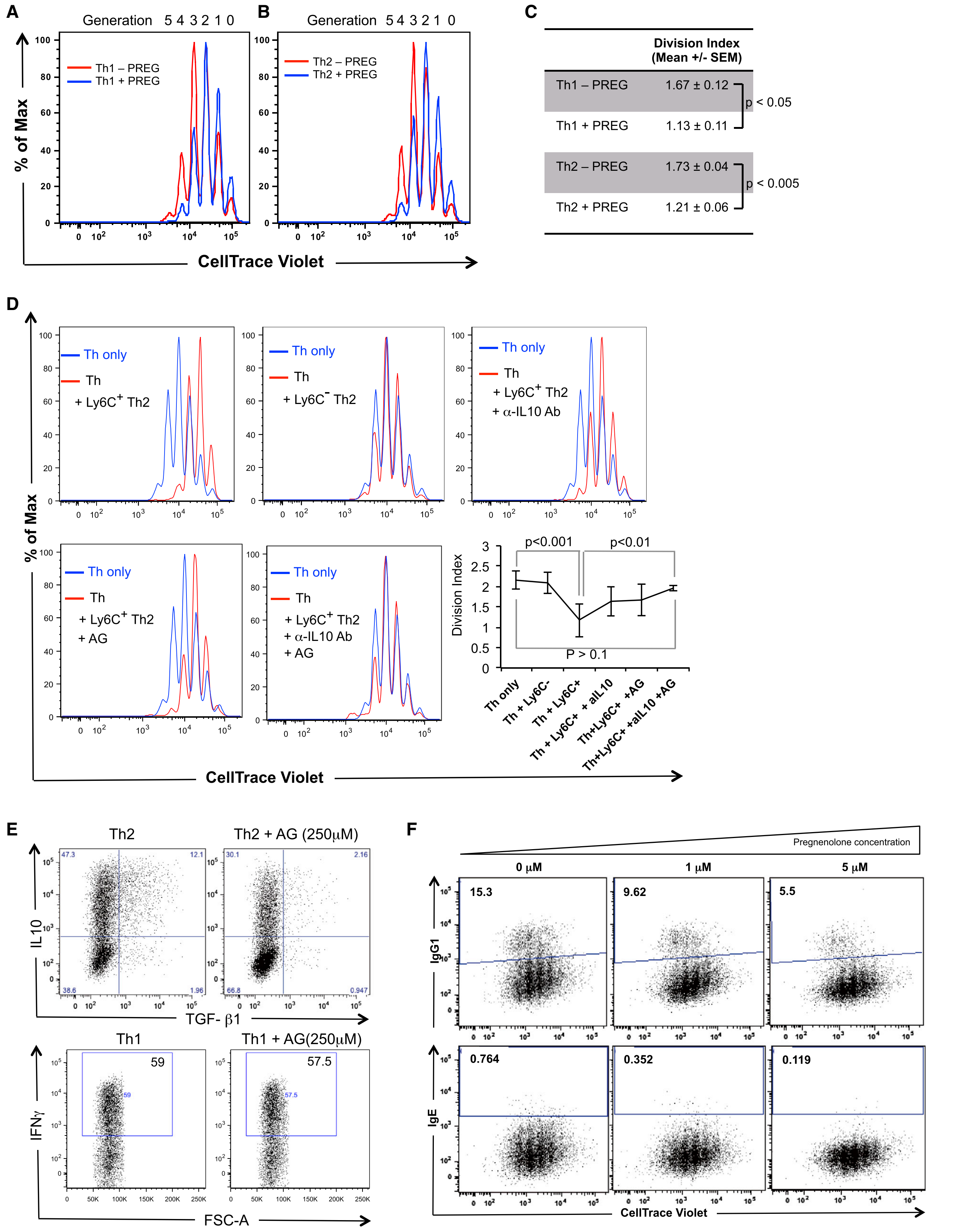

(legend on next page)

normal responder cell proliferation (blue histogram) (Figure S5B).

The results do not exclude the possibility of involvement of both pregnenolone and IL-10 in the same pathway, as we observed a significant inhibition of IL-10 and TGF-β1 production when Th2 cells were polarized in presence of AG (Figure 5E). In contrast, AG treatment had no effect on interferon (IFN)-γ production in Th1 cells, confirming the Th2 specificity (Figure 5E). Although we observed a correlation of TGF-β1 expression with Cyp11a1, neutralization of TGF-β1 had no effect on suppressor activity of Ly6C$^+$ cell, in contrast to IL-10 and pregnenolone (Figure S5A).

Pregnenolone Negatively Regulates In Vitro B Cell Differentiation

We studied the effect of pregnenolone on B cell class switch recombination using a well-established in vitro method (Uchimura et al., 2011). In brief, purified splenic B cells were stained with CellTrace Violet and allowed to differentiate in the presence of lipopolysaccharide (LPS) and IL-4 with or without pregnenolone. On day 5 of stimulation, cell-surface IgG1 and IgE were analyzed by flow cytometry. We observed a dose-dependent reduction of IgG1 and IgE-producing cells upon pregnenolone treatment (Figure 5F). The inhibition of class switching was independent of cell proliferation at the doses of pregnenolone we administered (Figure S5C).

DISCUSSION

This investigation revealed the transcriptional upregulation of key enzymes of the steroidogenic pathway during Th cell differentiation. Single-cell RNA sequencing provided us with high-resolution transcriptomic data, which revealed extensive heterogeneity within the Th2 population. We demonstrated the differential upregulation of Cyp11a1 in Th2 and showed that these cells are capable of de novo steroid synthesis, in vitro and in vivo.

Based on these observations, we propose the idea of a ''lymphosteroid,'' which can be defined as a steroid produced by a lymphocyte. We postulate that Th2 cells differentiate to steroid-producing Th2 cells in order to negatively regulate the type 2 immune response, possibly to restore homeostasis (Figure 6). Pregnenolone-dependent inhibition of two major type 2 events, the Th cell proliferation and B cell immunoglobulin class switching, support this idea. Our discovery of de novo steroid production in Th2 cells introduces the possibility of a steroid regulatory intracrine, autocrine, and/or paracrine network within the immune system.

Steroid hormones regulate the immune system, partly by cell-cycle inhibition, inhibiting effector immune cell differentiation and induction of cell death (Rhen and Cidlowski, 2005; Sakiani et al., 2013; Taylor et al., 2005). Steroids may exert their action by genomic pathways, involving hormone binding to specific nuclear receptors (NRs) and subsequent modulation of gene transcription after binding to DNA. Alternatively, these hormones can transduce signal in nongenomic ways without any direct interaction with chromatin, as reviewed in Lösel and Wehling (2003). In the context of immune responses, both the genomic and nongenomic pathways of steroid hormone actions may be operating.

Steroid production as a result of immune induction from mucosal tissues, such as in the lung and intestine, has been shown to play a tolerogenic role to maintain tissue homeostasis (Cima et al., 2004; Hostettler et al., 2012). It is interesting to note that intestinal mucosa and lung are target tissues of Th2-mediated immune response during helminthic infection and allergic asthma, respectively. Moreover, failure to induce lung steroid synthesis is asserted to be a cause of asthma and has been experimentally confirmed in the OVA-induced allergic airway inflammation model (Hostettler et al., 2012). It is possible that Th2 cells play a role in restoring type 2 immune homeostasis by producing a lymphosteroid, which can act directly or could be used as a precursor substrate by other cells (lung epithelial and intestinal epithelial) to metabolize other downstream bioactive steroids. Hence, our study suggests it is worth further investigating the contribution of steroid-producing Th2 cells in these contexts.

Interestingly, two recent publications have shown that Cyp11a1 is involved in the regulation of cytotoxic T cells in allergic lung disease and development of peanut-induced intestinal anaphylaxis (Jia et al., 2013; Wang et al., 2013). This

Figure 5. Immunosuppression by Pregnenolone and Pregnenolone-Producing Cells

(A–C) Pregnenolone inhibits Th cell proliferation. Naive Th cells were stained with CellTrace Violet and activated for Th1 (A) or Th2 (B) differentiation for 72 hr in the presence (blue histogram) or absence (red histogram) of pregnenolone (5 μM). The cell proliferation profile was captured by FACS based on dye decay. The histograms depicted are representative of three independent experiments with three to five mice each.

(C) Mean division indices were calculated for the experiments described in (A) and (B). Division index is the average number of divisions that a cell (present in the starting population) has undergone. p values were calculated using unpaired two-tailed t test.

(D) Ly6C$^+$ Th2 cells inhibit Th cell proliferation. CellTrace-Violet-stained naive Th cells (''responder cells'') were activated by anti-CD3e and anti-CD28 in the presence of FACS-sorted Ly6C$^+$ or Ly6C$^-$ Th2 cells and anti-IL-10 antibody and/or AG as indicated. The proliferation profile of the responder Th cells grown in the absence of Th2 (blue) was captured by FACS on the third day of activation. This was compared to the proliferation profile of responder Th cells activated in the presence of Th2 only (Ly6C$^+$ or Ly6C$^-$) (red). The Ly6C$^+$ cells were pretreated with neutralizing anti-IL-10 antibody (20 μg/ml) or with aminoglutethimide (AG, 250 μM) or with both for 24 hr and were used in the same culture conditions. The responder Th cell to Th2 (Ly6C$^+$ or Ly6C$^-$) cell ratio was 1:1. The histograms depicted are representative of three independent experiments with three mice in each experiment. Mean division indices ±SD are shown in the inset (right, bottom). p values were calculated using unpaired two-tailed t test.

(E) Inhibition of Cyp11a1 activity negatively regulates IL-10 and TGF-β1 expression in Th2. In vitro polarized Th2 and Th1 cells (3 days activation, 2 days resting) in the presence or absence of aminoglutethimide (AG, 250 mM) were FACS analyzed after reactivation with PDBU/Ionomycin for intracellular cytokine (IL-10 and TGF-β1 for Th2; IFN-γ for Th1) expression. Data shown are representative of three independent experiments.

(F) Effect of pregnenolone on B cell class switching to IgG1 and IgE. Naive resting B cells were stained with CellTrace Violet. Class switch recombination (CSR) was induced with LPS and IL-4 in the presence of different concentrations of pregnenolone. Cell-surface expression of IgG1 and IgE were analyzed by FACS on day 5 of stimulation. Data shown are representative of three independent experiments with three mice each. p values were calculated using unpaired two-tailed t test and were <0.05 when untreated groups were compared with 5 μM pregnenolone-treated groups.

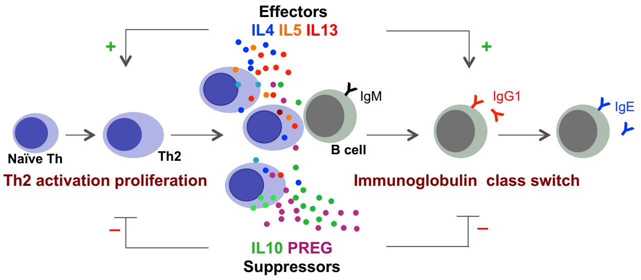

Figure 6. Proposed Model for Lymphosteroid Production by Cyp11a1⁺ Th2 Cells and Its Functional Role in Type 2 Immune Response

A subset of Th2 cells differentiates to become steroidogenic Th2 cells, which produce pregnenolone (PREG) as well as the suppressor cytokine IL-10. Together, these help to actively restore immune homeostasis, including through limiting Th cell proliferation and B cell differentiation.

supports the physiological relevance of de novo steroid production by immune cells.

EXPERIMENTAL PROCEDURES

Ethics Statement

All animal experiments were undertaken with the approval of the UK Home Office.

Th Cell Culture

Splenic naive Th cells were purified with the $CD4^+CD62L^+$ T Cell Isolation Kit II (Miltenyi Biotec) and polarized in vitro toward differentiated Th subtypes as described before in (Hebenstreit et al., 2011). In brief, naive cells were seeded into anti-CD3e (1 μg/ml, clone 145-2C11, eBioscience) and anti-CD28 (5 μg/ml, clone 37.51, eBioscience) -coated plates. The medium contained the following cytokines and/or antibodies for the different Th subtypes: Th1, recombinant murine IL-12 (10 ng/ml, R&D Systems) and neutralizing anti-IL-4 (5 μg/ml, clone 11B11, eBioscience); Th2, recombinant murine IL-4 (10 ng/ml, R&D Systems) and neutralizing anti-IFN-γ (5 μg/ml, clone XMG1.2, eBioscience); Th0, neutralizing anti-IL-4 (5 μg/ml, clone 11B11, eBioscience), neutralizing anti-IFN-γ (5 μg/ml, clone XMG1.2, eBioscience). The cells were removed from the activation plate on day 4. Th1 and Th2 cells were cultured for another four days in the absence of CD3 and CD28 stimulation. When required, cells were restimulated with phorbol dibutyrate and ionomycin (500 ng/ml, Sigma-Aldrich) for 4 hr in the presence of Monensin (2 μM, eBioscience) for the last 2 hr.

RNA-Seq Data Generation, Mapping, and Expression-Level Quantification

Poly(A)+ RNA was purified, reverse transcribed, and processed for sequencing as described previously (Hebenstreit et al., 2011, 2012) and in the Supplemental Experimental Procedures.

Single-Cell RNA Sequencing

Single-cell RNA-seq was performed following the methods described before in Brennecke et al. (2013) and in the Supplemental Experimental Procedures.

Quantitative PCR Analysis

Quantitative PCR analysis was performed as previously described (Mahata et al., 2012) and can be found in the Supplemental Experimental Procedures.

Western Blot Antibodies

Anti-CYP11A1 (Santa Cruz Biotechnology, C-16) and anti-TBP (Abcam) were used.

Quantitative Single-Cell Gene Expression Analysis by qPCR

Single-cell gene expression analysis was performed as described previously (Moignard et al., 2013) and can be found in the Supplemental Information.

Quantitative ELISA

Th cells were seeded and/or maintained with equal density, and supernatants were analyzed following manufacturer's protocol (Pregnenolone ELISA kit, Abnova). Th cell culture media used were with charcoal stripped fetal bovine serum (FBS) (Life Technologies and Invitrogen). Absorbance was measured at 450 nm, and data were analyzed in GraphPad Prism 5.

Extraction of Steroids and Quantification

Two milliliters of cell media with the addition of internal standard was extracted via liquid/liquid extraction using 4 ml of MTBE. The mixture was vortexed and then frozen, after which the MTBE layer was removed and evaporated at 55°C under nitrogen. The dried extract was subsequently reconstituted in 100 μl of 50/50 methanol/water before LC-MS/MS analysis. The samples were analyzed on a Waters Xevo Mass Spectrometer with an electrospray ionization source operated in positive mode. The liquid chromatography system attached was a Waters Acquity uPLC with a HSS T3, 1.8 μm, 1.2 × 50 mm column. The gradient system consisting of water with 0.1% formic acid and methanol with 0.1% formic acid was used. Pregnenolone was quantified by comparison to a calibration series ranging from 0.5 to 100 ng/ml with respect to the internal standard pregnenolone-d_4. The mass transitions for pregnenolone were 317.2 > 299.2 and 317.2 > 281.2 and for pregnenolone-d_4 was 321.2 > 303.2.

Nippostrongylus brasiliensis Infection

C57BL/6 mice were inoculated subcutaneously with 300 third-stage larvae. On day 5 or 10 postinfection, spleens and mesenteric lymph nodes were harvested. $CD4^+$ T cells were purified using MACS by depleting CD8a, CD11b, CD11c, Ly6G, and CD19-positive cells or selected positively with CD4 (L3T4) microbeads.

Ex Vivo Th Cell Culture for Pregnenolone ELISA

$CD4^+$ T cells (1×10^6) derived from *N. brasiliensis* infected or uninfected day 5 and day 10 mice were maintained in 200 μl charcoal stripped FBS containing Th cell-culture media for 24 hr without addition of any cytokines. Cell supernatants were analyzed for pregnenolone by ELISA (pregnenolone ELISA kit, Abnova).

FACS Analysis

In worm infection mouse model experiments, Th cells obtained on day 5 and day 10 postinfection from *N. brasiliensis* infected or control mice were analyzed by flow cytometry using anti-CD4-PE (eBioscience), anti-CYP11A1-fluorescein isothiocyanate (FITC) (mixture of Biorbyt and Santa Cruz), and anti-GATA3-Alexa Fluor 647 (eBioscience, Clone TWAJ) following the mouse regulatory T Cell staining kit protocol for FOXP3/transcription factors (eBioscience). Anti-CYP11A1 (Santa Cruz) antibody was conjugated to FITC using the Lynx rapid fluorescein antibody conjugation kit (AbD serotec) according to the manufacturer's instructions. Stained cells were analyzed on a FACSCalibur (BD Biosciences) flow cytometer using Cellquest Pro and FlowJo software.

In vitro Th cell experiments: staining was performed following eBioscience intracellular staining protocol for cytokines and nuclear staining/transcription factor staining protocol for different transcription factors (GATA3, FOXP3) and Cyp11a1, using eBioscience reagents and kits. The following antibodies were fluorescent dye-conjugated primary antibodies: IL-4, IL-13, IL-10, IFN-γ, TGF-β1, CD4, GATA3, FOXP3 (eBioscience); Ly6C, CD49b, LAG3, TCRb (BioLegend); Cyp11a1 (Bioss or mixture of Biorbyt and Santa Cruz). Stained cells were analyzed on a Fortessa (BD Biosciences) using FACSDiva and FlowJo software. CompBeads (BD Biosciences) were used for compensation where distinct positively stained populations were unavailable.

Th Cell Proliferation Assay

Naive Th cells were stained with CellTrace Violet following the CellTrace Violet Cell Proliferation Kit (Invitrogen) protocol and cultured under

activation-differentiation conditions for Th1 or Th2 as described previously but in the presence or absence of pregnenolone for 3 days. Flow cytometry was performed using an LSRII (BD) and data analysis with FlowJo software.

Th Cell Suppression Assay

Naive Th cells were stained with CellTrace Violet following the CellTrace Violet Cell Proliferation Kit (Invitrogen) protocol (responder cells) and cultured under activation conditions (plate coated with anti-CD3 and anti-CD28) in the presence or absence of FACS-sorted (Mo-Flo) Ly6C$^+$ or Ly6C$^-$ cells for 3 days. When required, Ly6C$^+$ cells were pretreated (24 hr) with anti-IL-10 (20 μg/ml), TGF-β1 neutralizing antibody (20 μg/ml), and/or aminoglutethimide (250 μM), and the responder cell growth condition was supplemented with similar reagents (i.e., anti-IL-10 neutralizing antibody and/or aminoglutethimide). Flow cytometry was performed using a Fortessa (BD Biosciences) and analyzing data with FlowJo software.

Immunoglobulin Class Switch Recombination

Splenic B cells from 8- to 12-week-old mice were purified by depletion of CD43$^+$ cells using anti-CD43-coupled magnetic beads (Miltenyi Biotec), stained with CellTrace Violet following the CellTrace Violet Cell Proliferation Kit (Invitrogen) protocol, seeded in 96-well plates (1×10^5 per well) in 200 μl RPMI supplemented with 10% FBS, 0.05 mM 2-mercaptoethanol, 25 ng/ml recombinant mouse IL-4 (R&D Systems), and 40 μg/ml LPS (Sigma-Aldrich). On day 3 or day 5 of stimulation, B cell Fc receptors were blocked with PBS containing 2% rat serum and 10 mM EGTA and stained with FITC-conjugated anti-IgG1 (BD Biosciences) PE-conjugated IgE (BioLegend). Flow cytometry was performed using an LSRII (BD), excluding dead cells by 7-AAD staining. Data were analyzed with FlowJo software.

ACCESSION NUMBERS

The ArrayExpress (https://www.ebi.ac.uk/arrayexpress/) accession number for the single-cell RNA sequencing data reported in this paper is E-MTAB-2512.

SUPPLEMENTAL INFORMATION

Supplemental Information includes Supplemental Experimental Procedures, five figures, and four tables and can be found with this article online at http://dx.doi.org/10.1016/j.celrep.2014.04.011.

ACKNOWLEDGMENTS

We would like to thank Alexander Betz, Tapio Lönnberg, and Andrew Deonarine for useful discussions, Helen E. Jolin for technical help with in vivo experiments; Padraic Fallon for providing *N. brasiliensis* larva; Maria Daly, Fan Zhang, Bee Ling, and William Cheng for help with flow cytometry; and Azim Surani for the use of the Fluidigm C1. We would also like to thank Mike Quail and Harold Swerdlow for help with barcoding and sequencing. We are grateful to the Lister Institute Research Prize, the European Research Council grant ThSWITCH (grant number: 260507), the Medical Research Council, the EMBL-EBI, and the Wellcome Trust Sanger Institute. X.Z. is supported by an Early Postdoc. Mobility Fellowship from Swiss National Science Foundation (SNSF, grant number PBELP2_143538). We dedicate this work to the memory of our late colleague Michael Neuberger.

Received: May 6, 2013
Revised: March 23, 2014
Accepted: April 3, 2014
Published: May 8, 2014

REFERENCES

Abbas, A.K., Murphy, K.M., and Sher, A. (1996). Functional diversity of helper T lymphocytes. Nature *383*, 787–793.

Altin, J.A., Goodnow, C.C., and Cook, M.C. (2012). IL-10+ CTLA-4+ Th2 inhibitory cells form in a Foxp3-independent, IL-2-dependent manner from Th2 effectors during chronic inflammation. J. Immunol. *188*, 5478–5488.

Angus, S.P., and Nevins, J.R. (2012). A role for Mediator complex subunit MED13L in Rb/E2F-induced growth arrest. Oncogene *31*, 4709–4717.

Barnes, P.J., and Adcock, I.M. (2003). How do corticosteroids work in asthma? Ann. Intern. Med. *139*, 359–370.

Baulieu, E.E., Robel, P., and Schumacher, M. (2001). Neurosteroids: beginning of the story. Int. Rev. Neurobiol. *46*, 1–32.

Blair, P.A., Noreña, L.Y., Flores-Borja, F., Rawlings, D.J., Isenberg, D.A., Ehrenstein, M.R., and Mauri, C. (2010). CD19(+)CD24(hi)CD38(hi) B cells exhibit regulatory capacity in healthy individuals but are functionally impaired in systemic Lupus Erythematosus patients. Immunity *32*, 129–140.

Brennecke, P., Anders, S., Kim, J.K., Kołodziejczyk, A.A., Zhang, X., Proserpio, V., Baying, B., Benes, V., Teichmann, S.A., Marioni, J.C., and Heisler, M.G. (2013). Accounting for technical noise in single-cell RNA-seq experiments. Nat. Methods *10*, 1093–1095.

Camberis, M., Le Gros, G., and Urban, J., Jr. (2003). Animal model of Nippostrongylus brasiliensis and Heligmosomoides polygyrus. Curr. Protoc. Immunol. *Chapter 19*, 12.

Carey, K.T., Tan, K.H., Ng, J., Liddicoat, D.R., Godfrey, D.I., and Cole, T.J. (2013). Nfil3 is a glucocorticoid-regulated gene required for glucocorticoid-induced apoptosis in male murine T cells. Endocrinology *154*, 1540–1552.

Cima, I., Corazza, N., Dick, B., Fuhrer, A., Herren, S., Jakob, S., Ayuni, E., Mueller, C., and Brunner, T. (2004). Intestinal epithelial cells synthesize glucocorticoids and regulate T cell activation. J. Exp. Med. *200*, 1635–1646.

Collison, L.W., and Vignali, D.A. (2011). In vitro Treg suppression assays. Methods Mol. Biol. *707*, 21–37.

Cua, D.J., Hinton, D.R., and Stohlman, S.A. (1995). Self-antigen-induced Th2 responses in experimental allergic encephalomyelitis (EAE)-resistant mice. Th2-mediated suppression of autoimmune disease. J. Immunol. *155*, 4052–4059.

Eckstrum, K., and Bany, B.M. (2011). Tumor necrosis factor receptor subfamily 9 (Tnfrsf9) gene is expressed in distinct cell populations in mouse uterus and conceptus during implantation period of pregnancy. Cell Tissue Res. *344*, 567–576.

Fallon, P.G., Jolin, H.E., Smith, P., Emson, C.L., Townsend, M.J., Fallon, R., Smith, P., and McKenzie, A.N. (2002). IL-4 induces characteristic Th2 responses even in the combined absence of IL-5, IL-9, and IL-13. Immunity *17*, 7–17.

Gagliani, N., Magnani, C.F., Huber, S., Gianolini, M.E., Pala, M., Licona-Limon, P., Guo, B., Herbert, D.R., Bulfone, A., Trentini, F., et al. (2013). Coexpression of CD49b and LAG-3 identifies human and mouse T regulatory type 1 cells. Nat. Med. *19*, 739–746.

Germain, R.N. (2008). Special regulatory T-cell review: A rose by any other name: from suppressor T cells to Tregs, approbation to unbridled enthusiasm. Immunology *123*, 20–27.

Gershon, R.K., and Kondo, K. (1971). Infectious immunological tolerance. Immunology *21*, 903–914.

Gorter, S.L., Bijlsma, J.W., Cutolo, M., Gomez-Reino, J., Kouloumas, M., Smolen, J.S., and Landewé, R. (2010). Current evidence for the management of rheumatoid arthritis with glucocorticoids: a systematic literature review informing the EULAR recommendations for the management of rheumatoid arthritis. Ann. Rheum. Dis. *69*, 1010–1014.

Gould, H.J., and Sutton, B.J. (2008). IgE in allergy and asthma today. Nat. Rev. Immunol. *8*, 205–217.

Hebenstreit, D., Fang, M., Gu, M., Charoensawan, V., van Oudenaarden, A., and Teichmann, S.A. (2011). RNA sequencing reveals two major classes of gene expression levels in metazoan cells. Mol. Syst. Biol. *7*, 497.

Hebenstreit, D., Deonarine, A., Babu, M.M., and Teichmann, S.A. (2012). Duel of the fates: the role of transcriptional circuits and noise in CD4+ cells. Curr. Opin. Cell Biol. *24*, 350–358.

Horiuchi, S., Onodera, A., Hosokawa, H., Watanabe, Y., Tanaka, T., Sugano, S., Suzuki, Y., and Nakayama, T. (2011). Genome-wide analysis reveals unique regulation of transcription of Th2-specific genes by GATA3. J. Immunol. *186*, 6378–6389.

Hostettler, N., Bianchi, P., Gennari-Moser, C., Kassahn, D., Schoonjans, K., Corazza, N., and Brunner, T. (2012). Local glucocorticoid production in the mouse lung is induced by immune cell stimulation. Allergy *67*, 227–234.

Jia, Y., Domenico, J., Takeda, K., Han, J., Wang, M., Armstrong, M., Reisdorph, N., O'Connor, B.P., Lucas, J.J., and Gelfand, E.W. (2013). Steroidogenic enzyme Cyp11a1 regulates Type 2 CD8+ T cell skewing in allergic lung disease. Proc. Natl. Acad. Sci. USA *110*, 8152–8157.

Keino, H., Takeuchi, M., Suzuki, J., Kojo, S., Sakai, J., Nishioka, K., Sumida, T., and Usui, M. (2001). Identification of Th2-type suppressor T cells among in vivo expanded ocular T cells in mice with experimental autoimmune uveoretinitis. Clin. Exp. Immunol. *124*, 1–8.

Kerdiles, Y.M., Stone, E.L., Beisner, D.R., McGargill, M.A., Ch'en, I.L., Stockmann, C., Katayama, C.D., and Hedrick, S.M. (2010). Foxo transcription factors control regulatory T cell development and function. Immunity *33*, 890–904.

Kraemer, F.B., Khor, V.K., Shen, W.J., and Azhar, S. (2013). Cholesterol ester droplets and steroidogenesis. Mol. Cell. Endocrinol. *371*, 15–19.

Lechner, O., Dietrich, H., Wiegers, G.J., Vacchio, M., and Wick, G. (2001). Glucocorticoid production in the chicken bursa and thymus. Int. Immunol. *13*, 769–776.

Lösel, R., and Wehling, M. (2003). Nongenomic actions of steroid hormones. Nat. Rev. Mol. Cell Biol. *4*, 46–56.

Mahata, B., Sundqvist, A., and Xirodimas, D.P. (2012). Recruitment of RPL11 at promoter sites of p53-regulated genes upon nucleolar stress through NEDD8 and in an Mdm2-dependent manner. Oncogene *31*, 3060–3071.

Marrack, P., Scott-Browne, J., and MacLeod, M.K. (2010). Terminating the immune response. Immunol. Rev. *236*, 5–10.

Midzak, A., Rone, M., Aghazadeh, Y., Culty, M., and Papadopoulos, V. (2011). Mitochondrial protein import and the genesis of steroidogenic mitochondria. Mol. Cell. Endocrinol. *336*, 70–79.

Miller, W.L., and Auchus, R.J. (2011). The molecular biology, biochemistry, and physiology of human steroidogenesis and its disorders. Endocr. Rev. *32*, 81–151.

Moignard, V., Macaulay, I.C., Swiers, G., Buettner, F., Schütte, J., Calero-Nieto, F.J., Kinston, S., Joshi, A., Hannah, R., Theis, F.J., et al. (2013). Characterization of transcriptional networks in blood stem and progenitor cells using high-throughput single-cell gene expression analysis. Nat. Cell Biol. *15*, 363–372.

Murphy, K., Travers, P., Walport, M., and Janeway, C. (2008). Janeway's Immunobiology, Seventh Edition (New York: Garland Science).

Myles, I.A., Fontecilla, N.M., Valdez, P.A., Vithayathil, P.J., Naik, S., Belkaid, Y., Ouyang, W., and Datta, S.K. (2013). Signaling via the IL-20 receptor inhibits cutaneous production of IL-1β and IL-17A to promote infection with methicillin-resistant Staphylococcus aureus. Nat. Immunol. *14*, 804–811.

Neill, D.R., Wong, S.H., Bellosi, A., Flynn, R.J., Daly, M., Langford, T.K., Bucks, C., Kane, C.M., Fallon, P.G., Pannell, R., et al. (2010). Nuocytes represent a new innate effector leukocyte that mediates type-2 immunity. Nature *464*, 1367–1370.

Ouyang, W., Liao, W., Luo, C.T., Yin, N., Huse, M., Kim, M.V., Peng, M., Chan, P., Ma, Q., Mo, Y., et al. (2012). Novel Foxo1-dependent transcriptional programs control T(reg) cell function. Nature *491*, 554–559.

Pletinckx, K., Döhler, A., Pavlovic, V., and Lutz, M.B. (2011). Role of dendritic cell maturity/costimulation for generation, homeostasis, and suppressive activity of regulatory T cells. Front Immunol *2*, 39.

Prussin, C., Yin, Y., and Upadhyaya, B. (2010). T(H)2 heterogeneity: Does function follow form? J. Allergy Clin. Immunol. *126*, 1094–1098.

Rhen, T., and Cidlowski, J.A. (2005). Antiinflammatory action of glucocorticoids—new mechanisms for old drugs. N. Engl. J. Med. *353*, 1711–1723.

Sakiani, S., Olsen, N.J., and Kovacs, W.J. (2013). Gonadal steroids and humoral immunity. Nat. Rev. Endocrinol. *9*, 56–62.

Schmidt, A., Oberle, N., and Krammer, P.H. (2012). Molecular mechanisms of treg-mediated T cell suppression. Front. Immunol. *3*, 51.

Shen, W.J., Zaidi, S.K., Patel, S., Cortez, Y., Ueno, M., Azhar, R., Azhar, S., and Kraemer, F.B. (2012). Ablation of vimentin results in defective steroidogenesis. Endocrinology *153*, 3249–3257.

Taylor, A.L., Watson, C.J., and Bradley, J.A. (2005). Immunosuppressive agents in solid organ transplantation: Mechanisms of action and therapeutic efficacy. Crit. Rev. Oncol. Hematol. *56*, 23–46.

Uchimura, Y., Barton, L.F., Rada, C., and Neuberger, M.S. (2011). REG-γ associates with and modulates the abundance of nuclear activation-induced deaminase. J. Exp. Med. *208*, 2385–2391.

Vacchio, M.S., Papadopoulos, V., and Ashwell, J.D. (1994). Steroid production in the thymus: implications for thymocyte selection. J. Exp. Med. *179*, 1835–1846.

Viganò, S., Perreau, M., Pantaleo, G., and Harari, A. (2012). Positive and negative regulation of cellular immune responses in physiologic conditions and diseases. Clin. Dev. Immunol. *2012*, 485781.

Wang, M., Ramirez, J., Han, J., Jia, Y., Domenico, J., Seibold, M.A., Hagman, J.R., and Gelfand, E.W. (2013). The steroidogenic enzyme Cyp11a1 is essential for development of peanut-induced intestinal anaphylaxis. J. Allergy Clin. Immunol. *132*, 1174.e8.

Zhu, J., and Paul, W.E. (2010). Heterogeneity and plasticity of T helper cells. Cell Res. *20*, 4–12.

Zhu, J., Yamane, H., and Paul, W.E. (2010). Differentiation of effector CD4 T cell populations (*). Annu. Rev. Immunol. *28*, 445–489.

Cell Reports, Volume *7*

Supplemental Information

Single-Cell RNA Sequencing Reveals T Helper Cells Synthesizing Steroids De Novo to Contribute to Immune Homeostasis

Bidesh Mahata, Xiuwei Zhang, Aleksandra A. Kolodziejczyk, Valentina Proserpio, Liora Haim-Vilmovsky, Angela E. Taylor, Daniel Hebenstreit, Felix A. Dingler, Victoria Moignard, Berthold Göttgens, Wiebke Arlt, Andrew N.J. McKenzie, and Sarah A. Teichmann

SUPPLEMENTAL INFORMATION

Figure S1

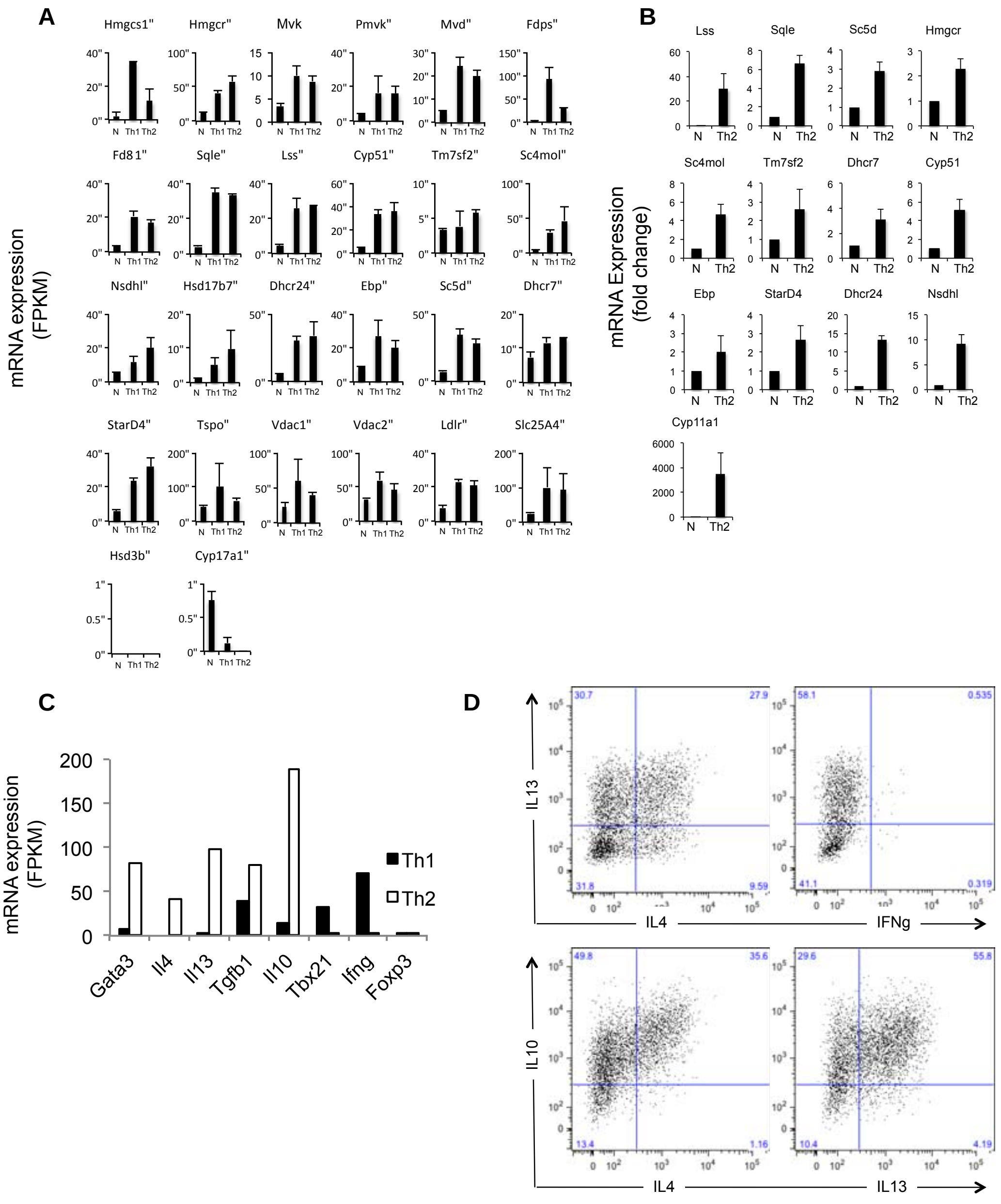

Figure S2

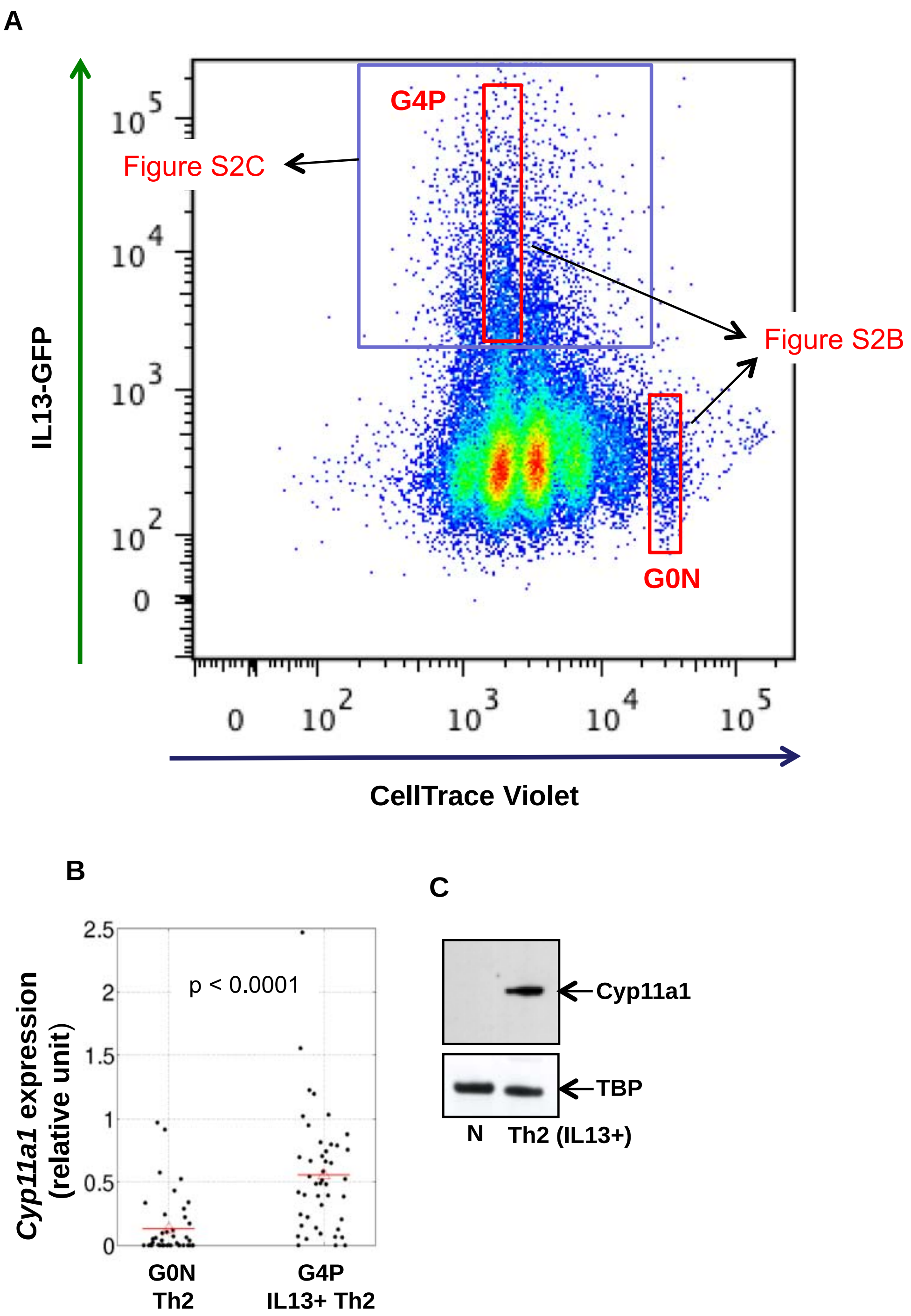

A
G4P
Figure S2C
Figure S2B
G0N
IL13-GFP
CellTrace Violet
B
p < 0.0001
Cyp11a1 expression (relative unit)
G0N Th2
G4P IL13+ Th2
C
Cyp11a1
TBP
N
Th2 (IL13+)

Figure S3

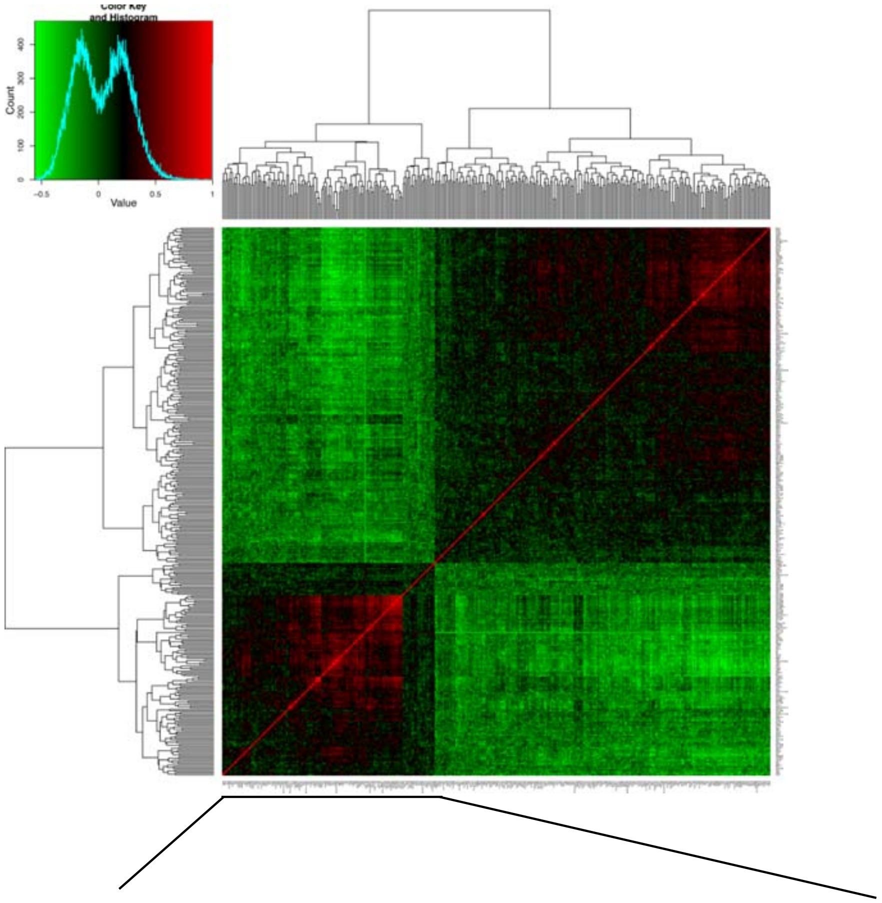

Gzma	Gpx8	Aldoart2	Ndufv3	Trav7−3
Mcf2l	Lst1	Aldoa	Sdf4	Pkp2
Tha1	Pik3cg	Aldoart1	Fxyd5	Gm16225
Fanci	Crygn	Gm8659	**Cyp11a1**	Ppp1r18
Gm16459	Gm13075	Whsc1l1	Gm11185	Gm12586
Rai2	Anxa2	Sell	GAPDH	Gm10658
Med13l	Ddit4	Samhd1	RP24−322E6.1	Sdc3
Syt2	Atp6v0d1	Nfil3	Gm7150	Nucb2
Gipr	Ostf1	Ctla2b	Gm3695	Lrrtm3
Lama5	0610010O12Rik	Ctla2a	Pgam1−ps2	Gcnt1
Kremen2	Anxa6	Ecm1	Tg	Hook2
Tsc22d4	Gm13668	Ly6c1	Gm7251	9430020K01Rik
Adamtsl4	Mir5115	Ly6c2	Gm11453	Tanc1
Icos	RP24−281K23.1	9030619P08Rik	Serpinf1	Zfyve16
Obfc1	Tmsb10	Ly6a	RP23−429I18.1	Gm8877
Cysltr2	Crip1	Basp1	Gm7293	Upf3a
Itgb3	Gm14925	Tgm2	Gm10293	Flywch1
Fam46a	Gm9790	Il12rb2	Gm12416	D19Wsu162e
Gm13914	Eif4ebp1	Mt1	Gm2308	Arfgap3
Gm13460	AC124553.1	Il24	AC168279.1	Slc6a20b
Dpm3	Ppp1r3b	Vim	Gm12286	
Sirt6	Ndrg1	Socs3	Gm5944	
Gm11550	Arrb1	Il4ra	Gm13394	
Gys1	5033411D12Rik	Cd24a	Gm13292	
Gm10767	Pold4	Tnfrsf9	Gm9164	
Afg3l2	Tpt1	Bsg	Gm15191	
Acbd7	S100a1	Fam162a	Gm10284	
Myo10	Pkm	Gm9843	Gm16348	
Rfc3	Gatsl3			

Figure S4

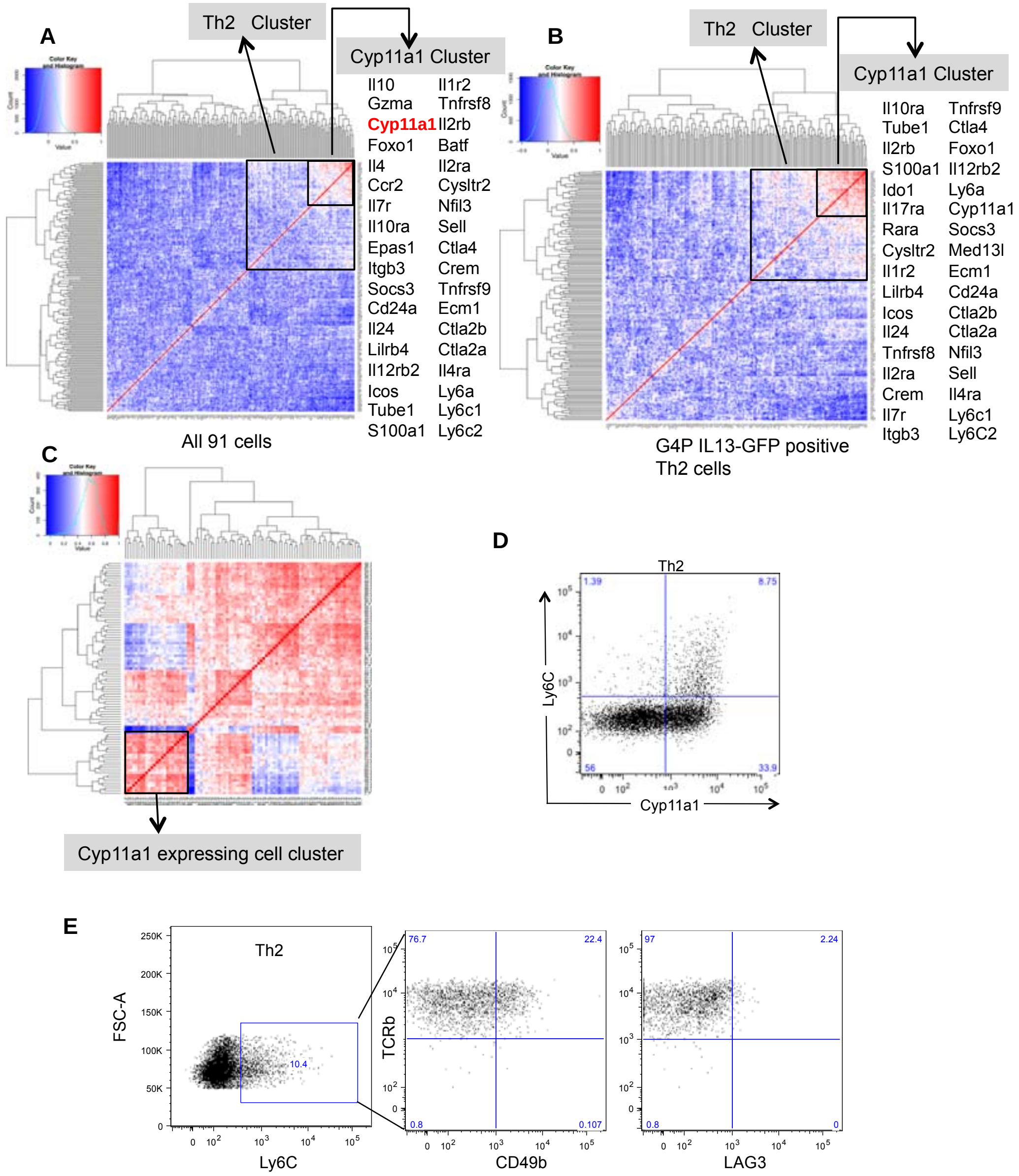

A
Th2 Cluster
Cyp11a1 Cluster
Il10 Il1r2
Gzma Tnfrsf8
Cyp11a1 Il2rb
Foxo1 Batf
Il4 Il2ra
Ccr2 Cysltr2
Il7r Nfil3
Il10ra Sell
Epas1 Ctla4
Itgb3 Crem
Socs3 Tnfrsf9
Cd24a Ecm1
Il24 Ctla2b
Lilrb4 Ctla2a
Il12rb2 Il4ra
Icos Ly6a
Tube1 Ly6c1
S100a1 Ly6c2
All 91 cells
B
Th2 Cluster
Cyp11a1 Cluster
Il10ra Tnfrsf9
Tube1 Ctla4
Il2rb Foxo1
S100a1 Il12rb2
Ido1 Ly6a
Il17ra Cyp11a1
Rara Socs3
Cysltr2 Med13l
Il1r2 Ecm1
Lilrb4 Cd24a
Icos Ctla2b
Il24 Ctla2a
Tnfrsf8 Nfil3
Il2ra Sell
Crem Il4ra
Il7r Ly6c1
Itgb3 Ly6C2
G4P IL13-GFP positive
Th2 cells
C
Cyp11a1 expressing cell cluster
D
Th2
Ly6C
Cyp11a1
1.39
8.75
56
33.9
E
Th2
FSC-A
Ly6C
10.4
TCRb
CD49b
76.7
22.4
0.8
0.107
LAG3
97
2.24
0.8
0

Figure S5

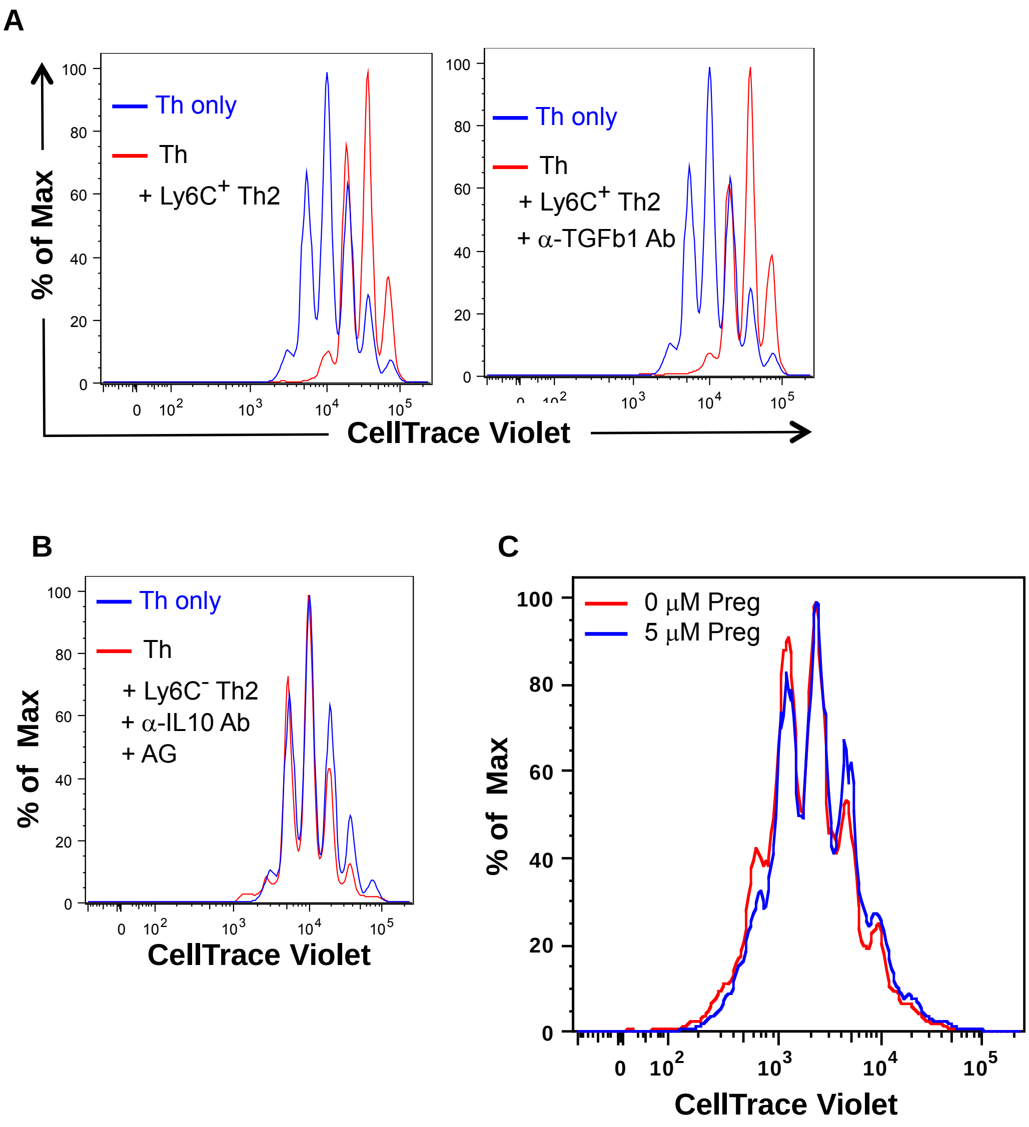
A
Th only
Th
+ Ly6C+ Th2
Th only
Th
+ Ly6C+ Th2
+ α-TGFb1 Ab
% of Max
CellTrace Violet
B
Th only
Th
+ Ly6C- Th2
+ α-IL10 Ab
+ AG
% of Max
CellTrace Violet
C
0 μM Preg
5 μM Preg
% of Max
CellTrace Violet

SUPPLEMENTAL FIGURE LEGENDS

Figure S1. Upregulation of a steroid synthesis pathway in Th2 cells *in vitro*

(A) Comparison of mRNA expression between Naïve (N), Th1 and Th2 cells

RNA-seq data for all genes involved in the steroid synthesis pathway was analyzed. Expression levels are shown as FPKM values. The histograms represent mean expression values for each mRNA obtained from two independent experiments. The values for naïve and Th2 are used in Figure 1 to generate color-coded maps. Please note that y-axis scales are variable, and expression levels at or below 1 FPKM are most likely simply stochastic gene expression as discussed in Hebenstreit et al., (2011).

(B) Quantitative PCR validation of selected genes in the steroid synthesis pathway. A set of genes observed to be upregulated in RNA-seq data were checked by qPCR. The bars represent mean expression values for each mRNA obtained from three independent experiments.

(C) RNA-seq gene expression profiles support the Th2-specific identity of cell culture. mRNA expression levels of Th2-specific genes Gata3, IL4, IL13; Th1-specific genes Tbx21 (or Tbet), IFNg; and Treg specific gene Foxp3. Expression levels are FPKM values in black for Th1 culture conditions and white for Th2. FPKM values are means obtained from two independent experiments.

(D) Cytokine expression profile of *in vitro* polarized Th2 cells by FACS after reactivation by PDBU and ionomycin.

Figure S2. Upregulation of Cyp11a1 in Th2 cells

(A) Gating strategy used to purify subpopulations of Th2 cells based on their cell division and IL13-GFP expression. Naïve T helper cells obtained from spleen of

IL13-GFP reporter mice were stained with CellTrace Violet dye and polarized for Th2 (3.5 days activation). The two subpopulations in the boxes were used in panels B and C below.

(B) Single-cell level comparison of Cyp11a1 mRNA expression between undifferentiated and differentiated Th2 cells. Cyp11a1 mRNA expression in single cells obtained from G0N and G4P subpopulations were detected by qPCR. Gene expression level was normalized to the mean of CD3e, Ubc, Hprt and Atp5a1 mRNA levels. Each dot represents a single cell. The two sets of values are significantly different (p-value 2.78×10^{-08}, Wilcoxon rank sum test).

(C) Splenic naïve Th cells from IL13-eGFP mice were activated-differentiated *in vitro* for Th2 polarisation. IL13-eGFP expressing mature Th2 cells were FACS sorted and analyzed for Cyp11a1 protein expression by Western blot.

Figure S3. Correlation heatmap of genes positively or negatively correlated with Cyp11a1 in single cell RNA-seq data. All genes with Spearman correlation coefficients with Cyp11a1 > 0.3 (133 genes) or < -0.3 (210 genes) were included for clustering based on the correlation coefficient. The bottom-left cluster corresponds to genes positively correlated with Cyp11a1 and the top-right cluster is negatively correlated genes.

Figure S4. Gene expression identity of Cyp11a1-expressing Th2 cells

(A,B,C) Cyp11a1 clusters with Th2 and suppressor genes and Cyp11a1+ cells cluster. (A) Hierarchical clustering on the Spearman correlation coefficient matrix of the 175 selected immunity genes (listed in Table S1) in all 91 cells. A large cluster

includes Th2 genes, and a more compact cluster includes Cyp11a1 and many suppressor genes.

(B) Hierarchical clustering on Spearman correlation coefficient matrix of the 175 genes in 52 G4P Th2 cells (4th generation and positive for IL13-GFP, Figure S4). A large cluster includes Th2 genes, and a more compact cluster includes Cyp11a1 with many suppressor genes.

(C) Hierarchical clustering on Spearman correlation matrix of cells based on 78 genes: 46 genes negatively correlated with Cyp11a1 (Spearman correlation < -0.35), and 32 genes positively correlated with Cyp11a1 (Spearman correlation > 0.35). The leftmost cluster (black box) of cells corresponds to those with high Cyp11a1 expression.

(D, E) Ly6C+ Th2 cells are steroidogenic and do not express type-1 regulatory (Tr1) markers LAG3 and CD49b

(D) *In vitro* polarized Th cells (3 days activated, 2 days resting) were FACS analyzed for Ly6C and Cyp11a1.

(E) *In vitro* polarized Th2 cells (3 days activated, 2 days resting) were analyzed for the presence of Ly6C, CD49b and LAG3 compared to the isotype control.

Figure S5.

(A) TGFb1 is not involved in Ly6C+ cell-mediated suppression. CellTrace Violet stained naïve Th cells were activated by anti-CD3e and anti-CD28 antibodies in the presence of FACS-sorted Ly6C+ or Ly6C- Th2 cells. The Ly6C+ cells were pre-treated with neutralizing anti-TGFb1 antibody (20μg/ml) for 24 hours. The histograms depicted are representative of 3 independent experiments with 3 mice in

each experiment. All culture conditions are as in Figure 5D. The first upper left panel of Figure 5D is reproduced here as the left panel for comparison.

(B) CellTrace Violet stained naïve Th cells ("responder cells") were activated by anti-CD3e and anti-CD28 antibodies in the presence of FACS sorted Ly6C- Th2 cells and anti-IL10 antibody and AG as indicated. The proliferation profile of the responder Th cells grown in the absence of Th2 (blue) was captured by FACS on the 3rd day of activation. This was compared to the proliferation profile of responder Th cells activated in the presence of Ly6C- Th2 (red). The Ly6C- cells were pre-treated with neutralizing anti-IL10 antibody (20µg/ml) or with aminoglutethimide (AG, 250µM) or with both, for 24 hours, and were used in the same culture conditions. The responder Th cell to Ly6C- Th2 cell ratio was 1:1. The panel is shown as a control for the same experimental condition when performed with Ly6C+ Th2 cells (as shown in Figure 5D, bottom row, right)

(C) No effect of pregnenolone on B cell proliferation. Proliferation of untreated and 5µM pregnenolone treated B cells was monitored by dye dilution in the same experiments described in Figure 6F. There is no significant difference between treated and untreated cells.

Table S1 List of 175 selected genes involved in immune response

Ahr	Cd86	Egr2	Il12a	Il5	Maf	Socs3
Asb2	Cd8b1	Egr3	Il12rb1	Il6	Med13l	Socs5
Batf	Cdkn1b	Epas1	Il12rb2	Il7	Nfatc1	Tanc2
Bcl6	Cebpb	F2r	Il13	Il7r	Nfatc2	Tbx21
Cblb	Cngb1	Foxo1	Il13ra1	Il9	Nfatc2ip	Tcf7
Ccl5	Crebbp	Foxp1	Il15	Inhba	Nfatc3	Tfcp2
Ccnjl	Crem	Foxp3	Il17ra	Irf1	Nfil3	Tgfb1
Ccr1	Csf1r	Gata3	Il17rb	Irf4	Nr1i2	Tgfb3
Ccr2	Csf2	Gfi1	Il18	Irf8	Nt5e	Tgfbi
Ccr3	Ctla2a	Gpr44	Il18bp	Itch	Ostf1	Tgfbr3
Ccr4	Ctla2b	Gzma	Il18r1	Itga2	Pcgf2	Tmed1
Ccr5	Ctla4	Gzmb	Il1r1	Itgae	Prdm1	Tmtc2
Ccr7	Cxcr1	Havcr2	Il1r2	Itgam	Prf1	Tnf
Ccr8	Cxcr3	Icam1	Il2	Itgb3	Ptgir	Tnfrsf14
Cd24a	Cxcr4	Icos	Il21	Jdp2	Ptprc	Tnfrsf18
Cd27	Cxcr5	Ido1	Il22	Junb	Rara	Tnfrsf25
Cd28	Cxcr6	Ido2	Il23a	Klrb1	Rnf128	Tnfrsf4
Cd38	Cyp11a1	Ifng	Il23r	Klrd1	Rora	Tnfrsf8
Cd3e	Cysltr2	Ifngr1	Il24	Lag3	Rorc	Tnfrsf9
Cd4	Ddit3	Ifngr2	Il27ra	Lif	Runx3	Tnfsf11
Cd40	Dgka	Igsf6	Il2ra	Lilrb4	Rxra	Tnfsf4
Cd40lg	Dpp4	Ikzf2	Il2rb	Lrrc32	S100a1	Tube1
Cd44	Dusp4	Il10	Il3	Ly6a	Sell	Tyk2
Cd68	Ebi3	Il10ra	Il4	Ly6c1	Socs1	Xbp1
Cd80	Ecm1	Il10rb	Il4ra	Ly6c2	Socs2	Yy1

Table S1. A list of genes characteristic of different cell types or involved in immune response (including effector and suppressor function). This list was compiled from the literature covering transcription factors, cell surface markers (receptors), cytokines and chemokines. 175 of these were found to be expressed at least at basal level in the 91 single cell transcriptomes. These genes were used to generate the clusters in Figures S6A and B.

Table S2. Genes with Spearman correlation coefficients with Cyp11a1 > 0.3 or s<-0.3, based on the single cell RNA-Seq data of all 91 Th cells (as shown in Figure 3A). For each gene, we annotate it by category: cytokine, transcription factor (TF), or surface marker.

Table S3. List of genes positively or negatively correlated with Cyp11a1 (Spearman correlation coefficient), based on the single cell RNA-Seq data of all 91 Th cells (G2N and G4P, as shown in Figure 3A). Genes with correlation coefficients > 0.2 or < -0.2 are shown, together with *p*-values.

Table S4. List of genes positively or negatively correlated with Cyp11a1 (Spearman correlation coefficient) based on the single cell RNA-Seq data of the 52 G4P Th2 cells (as shown in Figure 3A). Genes with Spearman correlation coefficients >0.2 or > -0.2 are shown, together with *p*-values.

EXTENDED EXPERIMENTAL PROCEDURES:

Abbreviations used in the Figure 1: ACoA: acetyl – CoA, AaCoA: Acetoacetyl-CoA, HCoA: HMG-CoA, M: mevalonate, M5P: mevalonate-5P, M5PP: mevalonate-5PP, IsPP: isopentyl-PP, FPP: farnesyl-PP, Squa: squalene, 23Ox: 2,3 oxydosqualene, Lan: lanesterol, 44Di: 4,4 dimethyl, cholesta-8,14,24-trienol, 14De: 14-demethyl-lanosterol, 4MZC: 4-methylzymosterol-carboxylate, 3K4M: 3-keto-4-methyl-zymosterol, 4MZ: 4-methylzymosterol, Zym: zymosterol, Cho8: cholesta-8,en-3beta-ol, Lath: lathosterol, DeC: 7-dehydro cholesterol, Cho7: cholesta-7,24-dien-3beta-ol, 7DeD: 7-dehydro-desmosterol, Des: desmosterol, OMM: outer mitochondrial membrane, IMM: inner mitochondrial membrane, Mito. Matrix: mitochondrial matrix.

T helper Cell Culture

Splenic naïve Th cells were purified by using the CD4+CD62L+ T Cell Isolation Kit II (Miltenyi Biotec) and were resuspended in IMDM, 10% FCS, 2 μM L-glutamine, penicillin, streptomycin and 50μM β-mercaptoethanol (purity > 95%). Alternatively CD4+CD25- naive T helper cells were purified by depletion of lymphocytes stained with FITC conjugated anti-CD8 (eBio Clone 53-6.7), anti-CD11b (eBio clone M1/70), anti-CD11c (eBio clone N418), anti-Ly6G (eBio Clone RB6-8C5), anti-CD19 (BD Bio Clone 1D3), and anti-CD25 (BD Bio Clone 7D4), and anti-FITC microbeads (Miltenyi Biotec) by MACS. Cells were seeded into anti-CD3e (1μg/ml, clone 145-2C11, eBioscience) and anti-CD28 (5μg/ml, clone 37.51, eBioscience) coated 96-well plates at a density of 250,000 to 500,000 cells/ml and a total volume of 200 μl. The medium contained the following cytokines and/or antibodies for the

different CD4+ subtypes. Th1: recombinant murine IL12 (10ng/ml, R&D Systems) and neutralizing anti-IL4 (5µg/ml, clone 11B11, eBioscience); Th2: recombinant murine IL4 (10ng/ml, R&D Systems) and neutralizing anti-IFN-γ (5µg/ml, clone XMG1.2, eBioscience); Th0: neutralizing anti-IL4 (5µg/ml, clone 11B11, eBioscience), neutralizing anti-IFN-γ (5µg/ml, clone XMG1.2, eBioscience). The cells were removed from the activation plate on day 4. Th1 and Th2 cells were cultured for another four days in absence of CD3 and CD28 stimulation to rest them. When required cells were restimulated with phorbol dibutyrate and ionomycin (both used at 500 ng/ml, both from Sigma) for 4 h in the presence of Monensin (2 µM, eBioscience) for the last 2 h after the resting phase (Hebenstreit et al., 2011).

RNA-seq, data generation, read mapping and expression level quantification

Poly-(A)+ RNA was purified from ~500 000 cells using the Oligotex kit (Qiagen). The manufacturer's protocol was slightly modified to include additional final elution steps resulting in a larger volume. After precipitation of RNA to concentrate it, first- and second-strand cDNA synthesis was performed using the Just cDNA kit (Stratagene), skipping the blunting step and directly proceeding to PCI extraction. Quality of the cDNA was tested by real-time PCR for a housekeeping gene. After this, the cDNA was sonicated for a total of 45 min using the Diagenode Bioruptor at maximum power settings, cycling 30 s sonications with 30 s breaks. Sequencing for 36 or 41bp was carried out on an Illumina GAII. The data were deposited at Gene Expression Omnibus (GEO, http://www.ncbi.nlm.nih.gov/geo/), accession numbers GSE28666 (Th2) and GSE31555 (all other sets). The reads for two biological replicates for each subtype were mapped using the spliced-read mapper TopHat version 1.2.0 (Trapnell et al., 2009) utilizing the Bowtie (version 0.12.7) short read

aligner (Langmead et al., 2009). The TopHat parameters used were "-r 328 –butterfly-search –solexal1.3-quals –p 8" and the "M. musculus, UCSC mm9" pre-built index file supplied by the TopHat website was used. To quantify expression levels, fragments per kilobase million (FPKM), reads per kilobase million (RPKM). Using Cufflinks version 0.9.3 (Trapnell et al., 2010), FPKM was calculated using the parameters "–reference-seq mm9.fa –GTF ucsc.gtf" where reference-seq enables bias correction, and –GTF looks for known transcripts in a gene transfer file (GTF) relative to the ucsc.gtf file. Cufflinks utilizes the maximum likelihood estimation method to calculate expression levels concurrently with various sequence and position specific biases which may affect expression levels, and produces a bias-adjusted measure (FPKM) of expression. A RefSeq based gene transfer file downloaded from the UCSC Genome Browser (accessed April 2011 from http://genome.ucsc.edu/ using the "table" function) was supplied to Cufflinks. Counts were determined from the TopHat SAM output using the HTSeq-counts program 0.4.7p4 (http://www-huber.embl.de/users/anders/HTSeq/doc/overview.html) using the parameters "–stranded=no --quiet." Counts were also used to calculate reads per kilobase million (RPKM) for single and paired end reads.

Quantitative PCR analysis

Total RNA was purified with the SV total RNA isolation kit (Promega) protocol. cDNA was prepared by annealing 500ng or 1μg RNA with oligo dT primers as per manufacturer's instructions (Transcriptor High Fidelity cDNA Synthesis kit, Roche). The cDNA samples were diluted 10 times with H_2O. 2 μl of diluted cDNA were used in 12μl qPCR reactions with appropriate primers and SYBR Green PCR Master Mix

(Applied Biosystems). Experiments were performed at least 3 times and data represent mean values +/- standard deviation.

Quantitative single-cell gene expression analysis

Single-cell gene expression analysis was performed using BioMark 96.96 Dynamic Array platform (Fluidigm, San Francisco, CA) and TaqMan Gene Expression Assays (Applied Biosystems, Carlsbad, CA). Single cells were sorted into 5µl of CellsDirect reaction mix and immediately stored in -80^0 C. Control wells containing 0 cells were included. On thawing, a mix containing 2.5µL gene specific 0.2x TaqMan gene expression assays (Applied Biosystems), 1.2 µL CellsDirect RT/Taq mix, and 0.3 µL TE buffer were added to each well. RT–PCR pre-amplification cycling conditions were: 50°C, 15min; 95°C, 2min; 22×(95°C, 15s; 60°C, 4min). Samples were diluted 1:5 in TE buffer and 6% were mixed with TaqMan Universal PCR Master Mix (Applied Biosystems). The sample mix and TaqMan assays were loaded separately into the wells of 96.96 Gene expression Dynamic Arrays (Fluidigm) in presence of appropriate loading reagents. The arrays were read in a Biomark analysis system (Fluidigm). ΔCt values were calculated in reference to the average of Atp5a1, Hprt1 and Ubc.

Single cell RNA sequencing and analysis:

Cell capture and library preparation for mouse cells using the Fluidigm C1 system

In vitro generated CellTrace Violet stained Th2 cells from 4th day of activation were FACS sorted to purify 4th generation IL13-GFP positive (G4P) and 2nd generation IL13-GFP negative (G2N), and mixed in equal proportion. 2000 cells were loaded onto a 10-17 micron C1 Single-Cell Auto Prep IFC, Fluidigm and cell capture was performed according to the manufacturer's instructions. The capture efficiency was

inspected using a microscope, and there were single cells in 93 positions and two cells in 3 positions. These 3 positions were noted and subsequently the data from these cells were removed from analysis. Upon capture, reverse transcription and cDNA preamplification were performed in the 10-17 micron C1 Single-Cell Auto Prep IFC, Fluidigm using the SMARTer PCR cDNA Synthesis kit (Clontech) and the Advantage 2 PCR kit. 1 µl of the ERCC Spike-In Control Mix (Ambion) in a 1:400 dilution in C1 Loading Reagent was added to the Lysis Mix. cDNA was harvested and diluted to a range 0.1-0.3 ng/µl and Nextera libraries were prepared using the Nextera DNA Sample Preparation Kit and the Nextera Index Kit (Illumina) following the instructions in the Fluidigm manual "Using the C1TM Single-Cell Auto Prep System to Generate mRNA from Single Cells and Libraries for Sequencing". Libraries were pooled and paired end 75 bp sequencing was performed on 8 lanes of an Illumina HiSeq.

Mapping of reads and normalization for the mouse data set (91 cells)

Paired-end reads from 93 samples containing single cells were mapped simultaneously to the Mus musculus genome (Ensembl version 38.70) and the ERCC sequences using GSNAP (version 2013-02-05)17 with default parameters. Two cells were removed at this stage due to very low numbers of reads mapping to these libraries, leaving 91 cells in total. From here we proceeded as described previously in Online Methods Steps 8-11 (Brennecke et al., 2013).

SUPPLEMENTAL REFERENCES:

Brennecke, P., Anders, S., Kim, J.K., Kolodziejczyk, A.A., Zhang, X., Proserpio, V., Baying, B., Benes, V., Teichmann, S.A., Marioni, J.C., *et al.* (2013). Accounting for technical noise in single-cell RNA-seq experiments. Nature methods.

Hebenstreit, D., Fang, M., Gu, M., Charoensawan, V., van Oudenaarden, A., and Teichmann, S.A. (2011). RNA sequencing reveals two major classes of gene expression levels in metazoan cells. Molecular systems biology *7*, 497.

Langmead, B., Trapnell, C., Pop, M., and Salzberg, S.L. (2009). Ultrafast and memory-efficient alignment of short DNA sequences to the human genome. Genome biology *10*, R25.

Trapnell, C., Pachter, L., and Salzberg, S.L. (2009). TopHat: discovering splice junctions with RNA-Seq. Bioinformatics *25*, 1105-1111.

Trapnell, C., Williams, B.A., Pertea, G., Mortazavi, A., Kwan, G., van Baren, M.J., Salzberg, S.L., Wold, B.J., and Pachter, L. (2010). Transcript assembly and quantification by RNA-Seq reveals unannotated transcripts and isoform switching during cell differentiation. Nature biotechnology *28*, 511-515.
